# Patterns of amygdala region pathology in LATE-NC: subtypes that differ with regard to TDP-43 histopathology, genetic risk factors, and comorbid pathologies

**DOI:** 10.1007/s00401-022-02416-5

**Published:** 2022-04-02

**Authors:** Matthew D. Cykowski, Anithachristy S. Arumanayagam, Suzanne Z. Powell, Andreana L. Rivera, Erin L. Abner, Gustavo C. Roman, Joseph C. Masdeu, Peter T. Nelson

**Affiliations:** 1 Department of Pathology and Genomic Medicine, Houston Methodist Hospital, Houston, TX 77030 USA; 2 Methodist Neurological Institute Department of Neurology, Houston Methodist Hospital, Weil Cornell Medicine, Houston, TX 77030 USA; 3 Sanders-Brown Center On Aging, University of Kentucky, University of Kentucky, Lexington, KY 40536 USA; 4 Department of Epidemiology, University of Kentucky, Lexington, KY 40536 USA; 5 Nantz National Alzheimer Center, Houston Methodist Hospital, Houston, TX 77030 USA; 6 Department of Pathology, University of Kentucky, Lexington, KY 40536 USA

**Keywords:** *GRN*, PART, Neuropathology, Tauopathy, Preclinical, Nondemented, DLB, Lewy

## Abstract

Transactive response (TAR) DNA-binding protein 43 kDa (TDP-43) pathology is a hallmark of limbic-predominant age-related TDP-43 encephalopathy (LATE). The amygdala is affected early in the evolution of LATE neuropathologic change (LATE-NC), and heterogeneity of LATE-NC in amygdala has previously been observed. However, much remains to be learned about how LATE-NC originates and progresses in the brain. To address this, we assessed TDP-43 and other pathologies in the amygdala region of 184 autopsied subjects (median age = 85 years), blinded to clinical diagnoses, other neuropathologic diagnoses, and risk genotype information. As previously described, LATE-NC was associated with older age at death, cognitive impairment, and the *TMEM106B* risk allele. Pathologically, LATE-NC was associated with comorbid hippocampal sclerosis (HS), myelin loss, and vascular disease in white matter (WM). Unbiased hierarchical clustering of TDP-43 inclusion morphologies revealed discernable subtypes of LATE-NC with distinct clinical, genetic, and pathologic associations. The most common patterns were: Pattern 1, with lamina II TDP-43 + processes and preinclusion pathology in cortices of the amygdala region, and frequent LATE-NC Stage 3 with HS; Pattern 2, previously described as type-β, with neurofibrillary tangle-like TDP-43 neuronal cytoplasmic inclusions (NCIs), high Alzheimer’s disease neuropathologic change (ADNC), frequent *APOE* ε4, and usually LATE-NC Stage 2; Pattern 3, with round NCIs and thick neurites in amygdala, younger age at death, and often comorbid Lewy body disease; and Pattern 4 (the most common pattern), with tortuous TDP-43 processes in subpial and WM regions, low ADNC, rare HS, and lower dementia probability. TDP-43 pathology with features of patterns 1 and 2 were often comorbid in the same brains. Early and mild TDP-43 pathology was often best described to be localized in the “amygdala region” rather than the amygdala proper. There were also important shared attributes across patterns. For example, all four patterns were associated with the *TMEM106B* risk allele. Each pattern also demonstrated the potential to progress to higher LATE-NC stages with confluent anatomical and pathological patterns, and to contribute to dementia. Although LATE-NC showed distinct patterns of initiation in amygdala region, there was also apparent shared genetic risk and convergent pathways of clinico-pathological evolution.

**Supplementary Information:**

The online version contains supplementary material available at 10.1007/s00401-022-02416-5.

## Introduction

The amygdala region comprises an anatomically heterogeneous set of brain structures that normally play a role in cognitive function, emotional processing, integration of olfactory input, and autonomic responses [2, 33, 54]. Consistent with its many functional roles, this brain region is anatomically complex [48]. The amygdala region consists of the corticomedial and basolateral subdivisions of the amygdala itself (the “amygdala proper”) [54], the temporal horn of the lateral ventricle and adjacent white matter and vasculature, the ventrolateral extension of the basal forebrain [15], the ventral extensions of putamen and claustrum, periallocortex (transentorhinal and entorhinal cortex), and neocortical regions of the inferior temporal lobe that are lateral to the collateral sulcus [7, 9, 35].

The amygdala region appears particularly susceptible to neurodegenerative disease pathology [40]. This brain area is implicated in early-stage tau, α-synuclein, and TAR DNA-binding protein 43 kDa (TDP-43) proteinopathies [9, 14, 31, 40]. TDP-43 pathology is one of the most common age-related pathologies [6, 23, 24, 27], initially described in patients with frontotemporal lobar degeneration (FTLD) and amyotrophic lateral sclerosis (ALS) [12, 34, 44], and thereafter recognized in other diseases [3, 5, 6, 11, 29, 51]. TDP-43 pathology can be observed in a presumed preclinical phase, but overall this pathologic biomarker is strongly associated with cognitive impairment independent of comorbid pathologies (e.g., Alzheimer’s disease-associated neuropathology, or ADNC) [29, 41, 53]. To aid in the recognition and classification of TDP-43 pathology in aging, a 2019 consensus working group proposed the term “limbic-predominant age-related TDP-43 encephalopathy”, or LATE [42].

The amygdala has been reported to be the earliest anatomic location of TDP-43 pathology in aging (LATE-NC Stage 1) [28, 39, 42]. In ground-breaking work, Josephs and colleagues described two patterns of TDP-43 proteinopathy in the amygdala, terming those patterns “type-β”, for fibrillary TDP-43 pathology associated with neurofibrillary tangles, and “type α”, for typical TDP-43 pathology in the form of neuronal inclusions and/or neurites [27]. Following these studies, important questions remain with respect to LATE neuropathologic change (LATE-NC) in amygdala and surrounding structures. Do the patterns of TDP-43 pathology overlap in individual brains? Are there other TDP-43 pathologic patterns, and if so, how do these patterns relate to comorbid pathologies and genetic risk factors? Does TDP-43 pathology in the amygdala begin in a predictable anatomic focus in all patients? Does LATE-NC ever begin outside of the amygdala per se (i.e., is it ever present in adjacent brain regions, but not in “amygdala proper”)? Does the involvement of the amygdala region at higher stages of LATE show convergence of TDP-43 pathology? Conversely, are there structures within the amygdala region more resistant to TDP-43 pathology even as pathologic burden increases? And finally, do all patterns of LATE-NC eventually result in higher-stage LATE-NC and dementia?

The aim of the current study was to enhance our understanding of LATE-NC heterogeneity, and to elucidate how TDP-43 pathologic patterns are associated with genetics, clinical disease severity, and comorbid pathologies. Pathologic features in the amygdala region were graded blind with respect to clinical diagnoses, genetic risk factors, and comorbid pathologies, and all included brains were assigned to TDP-43 pathologic pattern subtypes that were generated using an unbiased clustering algorithm. These patterns were then correlated with other parameters. Our findings build on prior studies about how TDP-43 pathology in LATE-NC originates and evolves in the aging brain.

## Materials and methods

### Case identification

Samples and data evaluated were from autopsy study participants from two different institutions: Houston Methodist Hospital (HMH, in Houston, Texas, USA) and the University of Kentucky Alzheimer’s Disease Research Center at Sanders-Brown Center on Aging (UKY [47], in Lexington, Kentucky, USA) (see Supplemental Table 1 (online resource)). Study participants were along a continuum from normal cognition to severe amnestic dementia. This was a convenience sample and there were no specific age cutoffs, but the demographics reflect the characteristics of the participants at each of the institutions. Research consent was provided for all study participants by the patients (and/or caregivers), or the next-of-kin at the time of autopsy consent, per the guidelines of each institution. The study was performed with the approval of the Institutional Review Boards at HMH and UKY.

### Exclusion criteria

Samples were excluded from participants with diagnoses of amyotrophic lateral sclerosis, clinical frontotemporal dementia with FTLD-TDP, FTLD-tau and other rare tauopathies (progressive supranuclear palsy, corticobasal degeneration), or other rare neurological diseases (e.g., prion disease). Samples were also excluded if review of anatomic sections showed poor orientation, incomplete sampling of the region, or acute hypoxic/ischemic injury.

### Histologic preparations and immunostaining

For evaluation of pathologic features in the amygdala region, formalin-fixed paraffin-embedded (FFPE) tissue blocks were sectioned at 6 µm, mounted on charged slides, and dried overnight at 60 °C. Serial sections were stained for hematoxylin and eosin (H&E) and cresyl violet/ luxol fast blue (LFB), using a previously described modified Klüver–Barrera protocol [40]. All including subjects and slides had appropriate amygdala region orientation, available clinical information, and whole-brain autopsy diagnoses.

All samples were stained for TDP-43 using phosphorylation-dependent (hereafter, “pTDP IHC”) and non-phosphorylation-dependent antibodies (hereafter, “TDP-43 IHC”), and were also stained for phospho-tau (AT8). The immunostaining procedure used has previously been described in detail [16, 22] and details are provided in the Supplemental Methods (online resource). Both TDP-43 antibodies were used as many laboratories with large brain banks rely on pTDP-43 IHC to detect TDP-43 proteinopathies [30] and TDP-43 IHC may be particularly sensitive to granular preinclusions [10, 13, 17], including in amygdala region [18].

### Amygdala region regions-of-interest

**Fig. 1:**
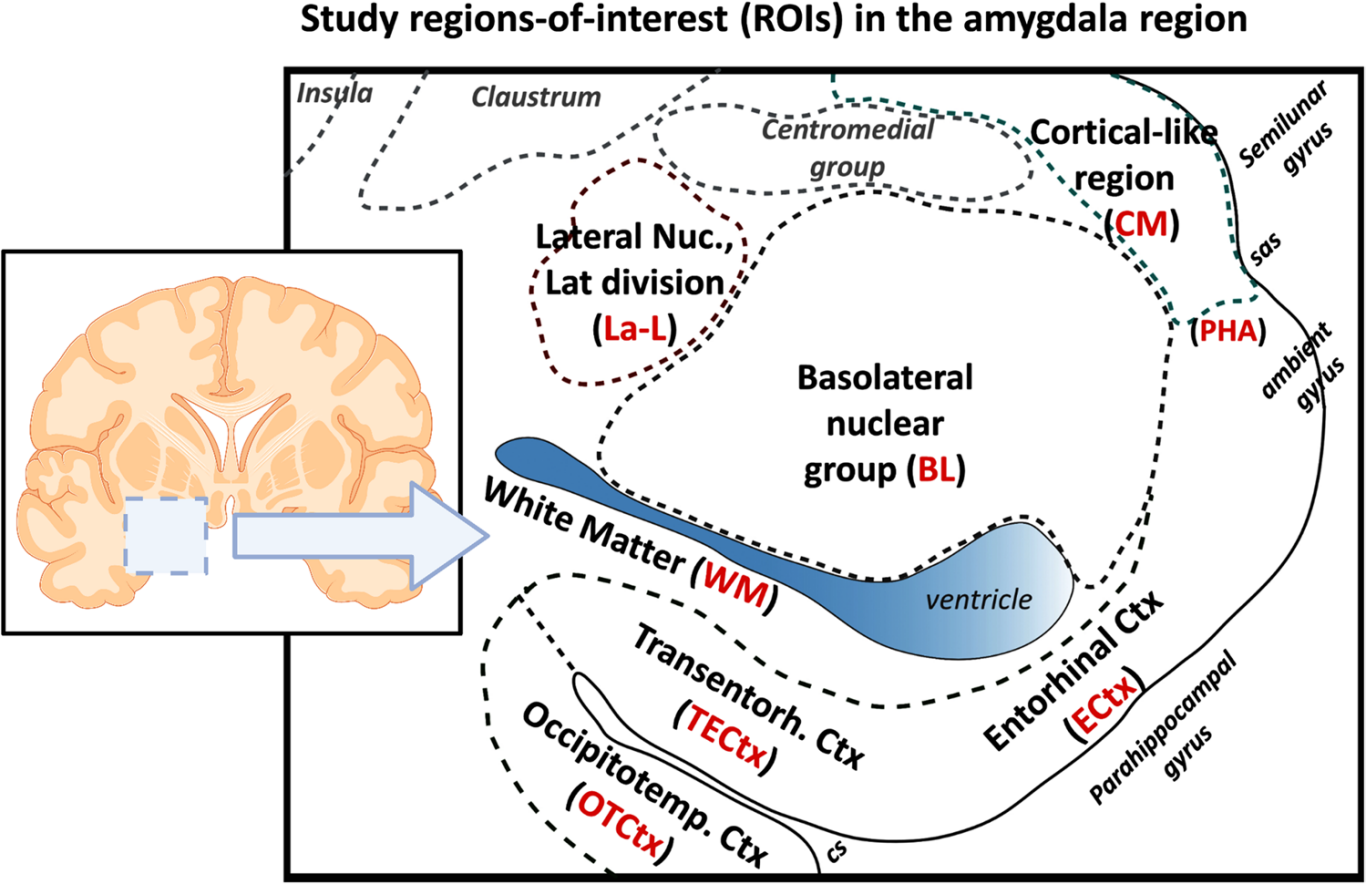
Regions-of-interest (ROIs) in the amygdala region. A schematic of the amygdala region and the associated ROIs is shown, with parahippocampal gyrus, collateral sulcus (cs) and semiannular sulcus (sas) labeled for reference. Study ROIs are in red, bolded font. Although not study ROIs, insula, ventral claustrum, centromedial nuclear group, and basal forebrain components were assessed for pTDP-43 pathology in all cases when present. Schematic created with BioRender

The regions-of-interest (ROIs) examined here are shown in Fig. 1, Supplemental Figs. 1 and 2 (online resource). ROIs included occipitotemporal cortex (*OTCtx*), transentorhinal and entorhinal cortex (*TECtx/ECtx*), corticomedial amygdala (*CM*), basolateral amygdala (*BL*), lateral subdivision of the lateral nucleus (*La-L*), and white matter (*WM*). Structures that were not consistently present across samples included the centromedial nuclear group—a dorsally located nuclear group in amygdala, the basal forebrain with magnocellular neurons of the nucleus basalis, the ventral extension of claustrum and putamen, and anterior hippocampus. However, pTDP and TDP-43 IHC pathologic observations were recorded when these structures were present.

The ROIs were defined as follows using H&E and LFB/Nissl-stained sections (Supplemental Fig. 1, online resource, which shows example study material). *OTCtx* was defined as neocortex of fusiform gyrus lateral to the collateral sulcus [7, 20, 27, 35]. *TECtx* and *ECtx* were defined here as used previously [9]. No attempt was made to separate *TECtx* and *ECtx* into two ROIs since large multipolar neurons in the pre-α layer of *ECtx* intercalate with laminae III and IV of *TECtx* [7]. The *CM* amygdala ROI included several fields in and around the uncus, including the subpial zone and molecular layer overlying amygdala, the underlying ventral cortical nucleus of amygdala containing several layers of pyramidal neurons [4], and the transition zone between ECtx and amygdala at the uncus, also known as the parahippocampal–amygdaloid transition area (PHA) [35]. *BL* amygdala represented the largest study ROI (Supplemental Figs. 1, 2, online resource), comprising large neurons of lateral, basal and accessory basal subnuclei, and scattered islands of small neurons (“intercalated nuclei”) [54]. *La-L* amygdala was the lateral-most ROI, very distinct as stripes of gray matter with medium-sized and large neurons and intervening white matter. The *WM* ROI focused on the white matter positioned between amygdala and entorhinal cortex. This *WM* ROI includes perivascular and periventricular white matter, enriched in corpora amylacea, veins with thickened and fibrotic walls (“venous collagenosis”), and rarefied white matter on LFB stain (Supplemental Fig. 3, online resource). This area, also referred to as “subamygdaloid white matter” [54], contains fiber bundles passing between the amygdala, entorhinal cortex, posterior parahippocampal gyrus, and hippocampus.

### Assessment of TDP-43 and pTDP-43 pathologies

**Fig. 2:**
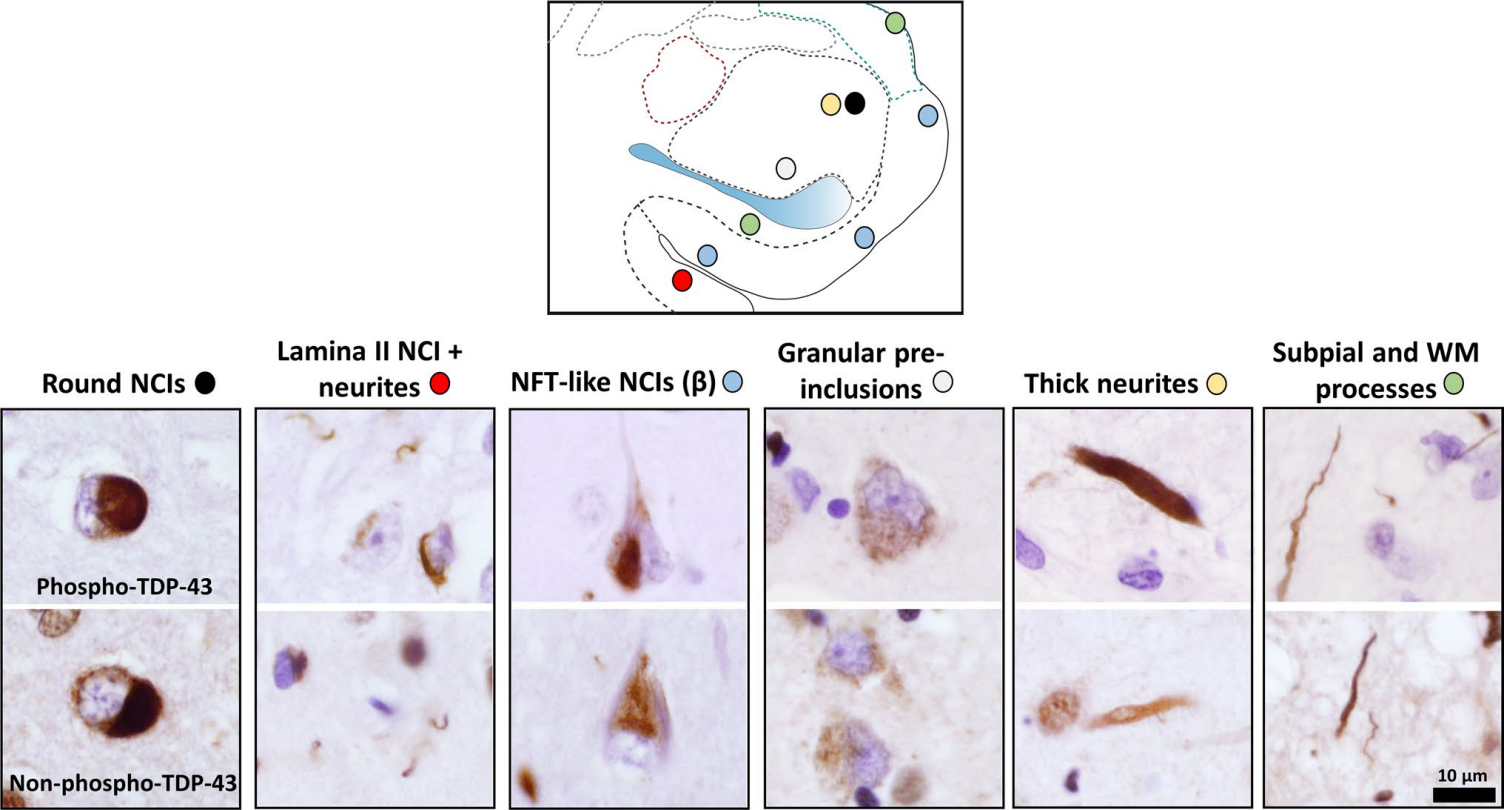
Key TDP-43 inclusion morphologies in LATE-NC. Six key TDP-43 inclusion morphologies are shown, with paired pTDP (top row) and TDP-43 IHC (bottom row). As shown, both pTDP and TDP-43 IHC can identify these pathologies. The colored circles adjacent to the morphology labels are placed on the Fig. 1 schematic to indicate their most common anatomic position in the amygdala region

TDP-43 IHC and pTDP IHC pathologies were scored blinded to clinical and pathologic data. Cases designated as “Positive” had inclusions in the same ROI by both TDP-43 and pTDP IHC. In addition, 6 morphologic subtypes of TDP-43 and pTDP IHC pathology were recorded for each participant as being present or absent. The 6 recorded morphologies were as follows:

(1) neurofibrillary tangle (NFT)-like neuronal cytoplasmic inclusions, consistent with type-β NCIs as previously described [27]; (2) round NCIs in amygdala; (3) small NCIs with non-tapering neurites in lamina II of cortex (FTLD-like); (4) granular preinclusions; (5) thick neurites in amygdala; and (6) tortuous processes in the subpial region of the CM ROI and the WM ROI. The typical appearance and location of each of these 6 key morphologies is shown in Fig. 2.

For each case, semiquantitative measures were applied to determine the severity of pTDP-43 pathology across the entire amygdala region and pTDP-43 pathologic burden within each ROI. The four-tiered semiquantitative scoring approach using pTDP-43 IHC is shown in Supplemental Fig. 4** (**online resource). Briefly, each amygdala sample was scored for pTDP-43 pathology as very mild pathology in 1 ROI (Score 1), multifocal and mild pathology in ≥ 2 ROIs (Score 2), multifocal and moderate pTDP-43 pathology (Score 3), and severe and extensive pathology (Score 4). For each ROI, pTDP-43 inclusion density was recorded in a representative 400 × field, recording 0–30 inclusions per 400 × field (maximum value was 30, as it is difficult to enumerate individual inclusions beyond that). We have previously demonstrated that TDP-43 IHC inclusion density assessed this way is strongly correlated with image-based analyses of pTDP IHC [13].

### Assessment of Tau pathologies

Phospho-tau (AT8) immunostaining was used to assess argyrophilic grain pathology [8, 26] and aging-related tau astrogliopathy (ARTAG) [31, 32], using standard criteria. ARTAG was recorded as being subpial, white matter and/or gray matter in location. NFT pathology was assessed and was concordant with ADNC pathology levels in all samples (these data were analyzed after blinded pathologic evaluations).

### Additional amygdala pathologies

Blinded to pTDP and TDP IHC data, H&E and LFB/Nissl stains were used to assess neuronal loss, vascular pathology, reactive astrocytosis, and myelin loss in the amygdala region, using FFPE sections obtained serially adjacent to those used for Tau, TDP-43, and pTDP IHC studies. The grading scheme for each of these pathologies is shown in Supplemental Fig. 5** (**online resource). A four-tiered scale was also used to rate small vessel arteriolosclerosis in the section as being absent, mild, moderate, or severe. Vascular pathology with rarefaction in subamygdaloid WM was rated as present (see example in Supplemental Fig. 3, online resource) or absent.

### Immunofluorescence studies

The immunofluorescence protocol used has been described in detail previously [16, 22] and is described in the Supplemental Methods (online resource). Briefly, a convenience sample of cases with TDP-43, pTDP, and tau pathology were selected for further examination using double immunofluorescence labeling. Cases were selected with prominent NFT-like (type-β) pTDP-43 NCIs, lamina II TDP-43 pathology, and subpial and WM pTDP-43 + processes. Double labeling was performed for Tau and non-phosphorylated TDP-43. Selected samples with prominent pTDP-43-positive processes were examined using double labeling with the axonal markers MAP-2, tubulin, and a pan-neuronal marker, as well as GFAP and CD44.

### Single nucleotide polymorphisms (SNP) genotyping

For SNP genotyping, DNA was isolated from FFPE samples using the QIAamp DSP DNA FFPE Tissue kit (Qiagen, catalogue ID 60,404, Germany) following manufacturer instructions. SNP genotyping was performed using TaqMan™ probes for *APOE* rs429358/ rs7412, *ABCC9* rs704180, *GRN* rs5848, *KCNMB2* rs12496790, and *TMEM106B* rs1990622 (Thermo Fisher Scientific, USA) on a Bio-Rad CFX 96 Real-Time System (Bio-Rad, USA). Interpretation was performed using the Allelic Discrimination function in CFX Maestro Software (Bio-Rad, USA). Further details of SNP genotyping are provided in the Supplemental Methods file (online resource).

### Clinical information and additional neuropathologic data

After pathologic data and genotyping were completed, additional clinical and pathologic data were added to the study database. Demographic and clinical data included participant age at death, sex, and cognitive status (normal, mild cognitive impairment, or dementia) proximal to death. Pathologic measures included brain weight, ADNC level (NIA/AA 2012) (“High”, “Intermediate”, “Low”, “Not”) [37], presence or absence of hippocampal sclerosis (HS), Lewy body disease (transitional/neocortical versus amygdala limited), presence or absence of primary age-related tauopathy (PART) [14], whole-brain arteriosclerosis (on a four-tied scale), Thal Aß phase [50], Braak NFT stage [9], and CERAD plaque frequency rating [36].

### Statistical analyses

Two-sided Wilcoxon ranked-sum tests and Chi-square (*χ*^2^) testing, as implemented in the software program R [49], were performed to examine group differences and associations of LATE-NC pathology versus the control group. The association between risk alleles and TPD-43 status was examined using the ‘epitools’ package in R [49]. The normal approximation (Wald) with small sample adjustment was used to determine odds ratio (OR) and confidence intervals. The publicly available tool Morpheus (https://software.broadinstitute.org/morpheus) was used to perform unbiased hierarchical clustering of LATE-NC + samples. Post hoc evaluation of group differences between clusters was performed using a non-parametric Kruskal–Wallis rank sum test, as implemented in R.

## Results

### Characteristics of the study cohort

The final study sample including *N* = 184 participants. Median age at death among included subjects was 85 years, median Braak NFT stage was III, and approximately one-third of patients were cognitively normal proximal to death. Demographic, clinical, and neuropathologic characteristics of the study cohort are summarized in Table 1. Additional study data are provided in Supplemental Table 1 (online resource).

**Table 1:** Characteristics of the study sample (*N* = 184)

Variable	Frequency/*N*	Median (IQR)/other
Age	–	85 years (11)
Male/female	48% / 52%	85 years (12) / 86 years (10.5)
Cognition^a^		
Dementia	51%	85 years (14)
Mild impairment	15%	89 years (7.5)
Normal	34%	84 years (9)
Brain weight	–	1200 gm (219)
Braak stage	–	NFT stage III (3)
Thal phase	–	Thal Phase 4 (2)
Arteriosclerosis (0–3)^b^	–	1, “Mild” (1)
ADNC level		
High	*N* = 59^c^	NFT Stage V (*N* = 26), VI (*N* = 33)^d^
Intermediate	*N* = 41	NFT Stage III (*N* = 18), IV (*N* = 13), V (*N* = 10)
Low	*N* = 41	NFT Stage I (*N* = 15), II (*N* = 19), III (*N* = 4), IV (*N* = 3)
Not	*N* = 23	NFT Stage 0 (*N* = 3), I (*N* = 10), II (*N* = 4), III (*N* = 4), IV (*N* = 2)
Lewy body pathology		
Transitional/diffuse	18%	NFT stage IV (2.8)
Absent	82%	NFT stage III (3)
Age-related pathology ^e^		
PART	18%	–
Argyrophilic grains	28%	–
ARTAG (any)	63%	–
Hippocampal sclerosis		
Present	26%	NFT stage V (2)
Absent	74%	NFT stage III (3)

^a^Cognitive status was unknown in 4 participants, and percentage shown for known samples

^b^For entire brain; see Methods for description

^c^Alzheimer’s Disease Neuropathologic Change (ADNC) level for known samples (total *N*)

^d^The number of Braak NFT stages for that ADNC level

^e^For PART, this included “Possible” and “Definite” categories. For ARTAG, this included any form (see text). AGD and ARTAG status were unknown for five participants (% of known samples is shown)

### Clinical, pathologic, and genetic comparisons between LATE-NC + and LATE- participants

Participants with LATE-NC (LATE-NC +) were older at death (*P* < 0.0001) and had a greater level of cognitive impairment (*P* < 0.02) (Table 2). Pathologically, LATE-NC + participants had a higher frequency of HS (P < 0.00001). Among amygdala region pathologies, LATE-NC + participants had higher rates of ARTAG (*P* < 0.0001), fibrillary astrocytosis (*P* < *0.001)*, WM vascular pathology (*P* < 0.05), and myelin and neuron loss (both *P* < 0.01) (Table 2).

**Table 2:** Clinical and pathologic differences between LATE+ and LATE− amygdalae

Variable^a^	LATE+ (*n* = 107)	LATE− (*n* = 77)	*P* value
Clinical			
Age	88 years (10.5) ^b^	83 years (11)	< *0.0001*
Cognitive impairment	74%	55%	< *0.02*
Male, female	50% Male, 50% Female	45% Male, 55% Female	NS
Whole brain pathology			
Hippocampal sclerosis	40%	5%	< *0.00001*
Transitional or diffuse LBD	21%	14%	NS
“High” ADNC	33%	42%	NS
NFT stage	NFT Stage IV (3)	NFT Stage III (3)	NS
Amygdala Region Pathology^c^			
ARTAG	76%	44%	< *0.0001*
Neuron loss, ECtx (0–3)	1 (2)	1 (1)	< *0.001*
Myelin loss (LFB) (0–3)	1 (2)	1 (1)	< *0.01*
Fibrillary astrocytosis ( ±)	71%	44%	< *0.001*
Venous collagenosis with rarefied WM ( ±)	54%	38%	< *0.05*
Argyrophilic grains	32%	24%	NS

Significant *P*-values are italicized

*NS* not significant; *ECtx* entorhinal cortex

^a^Determination of LATE+ and LATE− in amygdala region was made blinded to the variables shown in this table (e.g., ARTAG)

^b^Median value (IQR) are shown, unless otherwise specified

^c^Please see Methods for details

LATE-NC + and LATE-NC- participants did not significantly differ in the frequency of Lewy body disease, the frequency of high ADNC, or by NFT stage. The frequency of argyrophilic grains did not significantly differ between groups.

Among SNP genotypes examined, TDP-43 pathology was strongly associated with the presence of a risk allele (A) in *TMEM106B* rs1990622 (OR = 3.3, *P* = 0.003) (Table 3).

**Table 3:** SNP genotypes of LATE+ and LATE− amygdalae

Gene (SNP ID)	LATE+	LATE−	Odds ratio (CI)	*P* value
*TMEM106B* (rs1990622)				
Risk allele (A) carrier	90.5%	74.0%	3.3 (1.5–7.9)	*0.003*
*APOE* (rs429358, rs7412)				
Risk allele (ε4) carrier	36%	42%	0.79 (0.4–1.4)	NS
*GRN* (rs5848)				
Risk allele (T) carrier	50%	39%	1.6 (0.9–2.9)	NS
*ABCC9* (rs704180)				
Risk genotype (AA)	25.5%	24.0%	1.1 (0.5–2.2)	NS
*KCNMB2* (rs12496790)				
Risk genotype (AA)	7.8%	3.9%	2.0 (0.5–9.9)	NS

Significant *P*-values are italicized

### Patterns of LATE-NC in the amygdala region

**Fig. 3:**
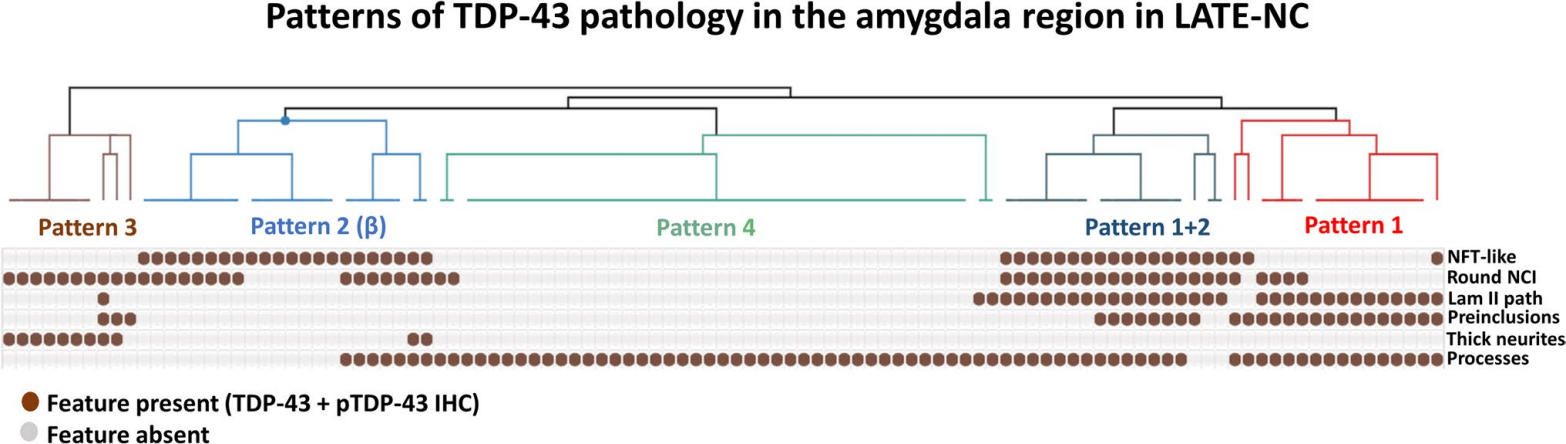
Patterns of TDP-43 pathology in the amygdala region of patients with LATE-NC (*N *= 107). Unbiased hierarchical clustering was performed in the publicly available program Morpheus (see Methods for detail). The resulting dendrogram is shown, and the TDP-43 morphologies shown to the right (“NFT-like”, “Round NCI”, etc.) are those illustrated in Fig. 2

As shown in Fig. 2, six key TDP-43 and pTDP morphologies were recorded for each case. It was common that multiple TDP-43 morphologic subtypes were present in a single brain, so individual brains could not be classified by a single feature alone. Therefore, these six morphologies were used in a clustering algorithm, resulting in patterns of LATE-NC that could be discerned in the resulting dendrogram (Fig. 3). These LATE-NC patterns are hereafter referred to as Patterns 1–4. An additional group had a common combination of Patterns 1 and 2 TDP-43 pathologies.

Pattern 1 was distinguished by prominent lamina II TDP-43 pathology in cortical sites of the amygdala region (OTCtx, TECtx, and ECTx), granular preinclusions, and processes in subpial and WM sites. Double labeling with Tau demonstrated these Lamina II inclusions did not co-localize with Tau (see Supplemental Fig. 6a, online resource). In any given microscopic field, the granular preinclusions in Pattern 1 histologically resembled those often seen in ALS and FTLD-TDP patients.

Brains designated as Pattern 2 samples had fibrillary, NFT-like TDP-43 NCIs, corresponding to the previous description of type-β by Josephs and colleagues [27]. These were most conspicuous in TECtx and ECtx. Round NCIs were variably seen in this pattern, and the junction between ECtx and amygdala (PHA) was a frequent site of involvement. Double labeling studies demonstrated that TDP-43 and Tau co-localized in NCIs of this pattern (see Supplemental Fig. 6b, online resource), as previously described [27].

A common combination of pathologies was seen with features of both Pattern 1 and Pattern 2 in the same brains. These participants had type-β NCIs (NFT-like), as well as small lamina II NCIs and neurites in cortical regions (OTCtx, TECtx, ECtx) (FTLD-like), and NCIs in amygdala. Granular preinclusions were variably seen, as were subpial and WM pTDP-43 immunoreactive processes.

Pattern 3 was characterized by round amygdala pTDP-43 NCIs and thick neurites in amygdala. Brains with this pattern mostly lacked other features of Patterns 1 and 2. pTDP-43 immunoreactive NCIs in Pattern 3 were seen in both CM and BL ROIs and thick neurites were more apparent on pTDP IHC than TDP-43 IHC staining.

Pattern 4 was the most frequent pattern in this study and was represented by subpial and/or WM TDP-43 + processes, often with no or rare NCIs. In these brains there were tortuous subpial TDP-43 + processes often wrapped around corpora amylacea. In WM, they could also be identified adjacent to thick-walled blood vessels, or in rarefied white matter. Double labeling studies demonstrated the TDP-43 processes were bounded by CD44-positive membranes (Supplemental Fig. 7c, online resource). CD44 highlights astrocytic membranes (Supplemental Fig. 7a, online resource), and the TDP-43 processes were in areas enriched for GFAP labeling (not shown), suggesting some component of this pathology is within astrocytes. The TDP-43 + processes did not co-localize exactly with tau pathology when ARTAG was also present (Supplemental Fig. 6c, online resource), although both pathologies could affect the same anatomic region. In addition, these TDP-43 + processes did not co-localize with the axonal markers MAP-2, Tubulin, or pan-neuronal marker (Supplemental Fig. 8, online resource).

### Comparing parameters between LATE-NC patterns

Table 4 shows how the identified LATE-NC patterns related to other parameters: patient age, LATE-NC stage, Braak NFT stage, ADNC level, presence of LBD, HS, and PART, frequency of the *APOE* ε4 allele, and frequency of normal cognition.

**Table 4:** Patterns of LATE-NC in the amygdala region based on hierarchical clustering

Pattern	Symbol^a^	TDP-43	Age	Frequency	Stage 1	Stage 2	Stage 3	NFT stage	No/Low ADNC	LBD^c^	PART^d^	Hipp Scl	APOE ε4	Normal cognition
**1**	α	Lamina II NCIs + short neurites, many granular preinclusions^b^	87.9	15%	13%	50%	**38%**	III	**63%**	6%	20%	**56%**	25%	38%
**2**	β	NFT-like NCIs, no/rare preinclusions	86.4	21%	**29%**	**67%**	5%	**V**	14%	27%	5%	**55%**	**64%**	18%
**1 + 2**	β + α	Features of Patterns 1 and 2	88.5	16%	**6%**	**76%**	18%	IV–V	18%	24%	0%	**82%**	50%	0%
**3**	γ	Round NCIs in amygdala + thick neurites	76.8	9%	**78%**	**11%**	11%	IV	20%	**60%**	0%	20%	50%	0%
**4**	δ	Subpial and WM processes, no/rare NCIs	86.2	39%	**62%**	**33%**	5%	**II**	**62%**	14%	**25%**	14%	17%	**44%**
		*P* value ^e^	**-**	**–**	**–**	**–**	< *0.001*	< *0.001*	< *0.0002*	*0.01*	*0.05*	< *0.001*	< *0.002*	< *0.002*

Notable values for the Patterns shown are indicated in bold type

*NS* not significant

^a^Proposed terminology (see Discussion)

^b^Granular preinclusions with loss of nuclear staining and accumulated cytoplasmic TDP-43, as seen in ALS and FTLD-TDP, using non-phosphorylated TDP-43

^c^Transitional and diffuse Lewy body disease

^d^Including both “Possible” and “Definite” PART

^e^*P* value from non-parametric ANOVA (Kruskal–Wallis rank sum test) of these variables, with cluster as the grouping variable. *TMEM106B* and *GRN* risk allele frequency did not significantly vary across clusters (data not shown)

Pattern 1 cases, which may be referred to as “type-α”, had the highest frequency of Stage 3 LATE. (The suggested use of type-α here relates to the use of type-α in work of Josephs et al*.* [27], though here indicating specifically Pattern 1, rather than any non-β TDP-43, as originally described in that work). Despite the superficially FTLD-TDP like lamina II pathology in periallocortical and neocortical regions of amygdala, these patients had one of the highest average ages at death (88 years), being much higher than is typical for FTLD-TDP. In addition, prior work has shown this group typically has less severe pTDP-43 IHC pathology in frontal cortices than is seen in FTLD-TDP [46]. Pattern 1 cases generally had only a limbic Braak NFT stage (III) and > 50% of the cases had “No” or “Low” ADNC. The *APOE* ε4 allele was not enriched in this group.

Pattern 2, which we designated “type-β” for consistency with Josephs et al*.* [27], had the highest levels of ADNC including highest median NFT stage. This group was enriched for the *APOE* ε4 allele (> 60%). In distinction from Pattern 1, this group had more frequent LATE-NC Stage 1, and especially LATE-NC Stage 2 cases.

The combination Pattern 1 + 2 group had a similar profile to Pattern 2, with higher ADNC and higher NFT stage, and was similarly enriched for Stage 2 LATE-NC and *APOE* ε4 (like Pattern 2 samples). Features of Pattern 1 were also present, including lamina II pTDP-43 NCIs. The rates of cognitive impairment (100%) and comorbid HS (82%) were greatest in this group.

Pattern 3 generally lacked features of Patterns 1 and 2, and instead had rounded amygdala NCIs. This pattern was most notable for the high frequency of transitional and diffuse LBD (60%) as compared to other pTDP-43 pathologic patterns (Table 4). Pattern 3 brains were predominantly LATE-NC Stage 1. Nonetheless, among persons with Pattern 3 samples, all had cognitive impairment.

Pattern 4 was the most prevalent LATE-NC group, constituting nearly 40% of all LATE-NC + samples in this study. Most participants with Pattern 4 were LATE-NC Stage 1. Further, normal cognition was most common in this group, and HS co-pathology was least common. This group had the lowest median NFT stage and the most frequent “No” or “Low” ADNC levels, as well as the highest rate of comorbid PART pathology. Building on the earlier use of “type α” and “type β” by Josephs and colleagues [27], we suggest the use of “type γ” and “type δ” for Patterns 3 and 4, respectively (see Table 4).

### Distribution of pTDP pathology in early and advanced LATE-NC stages

**Fig. 4:**
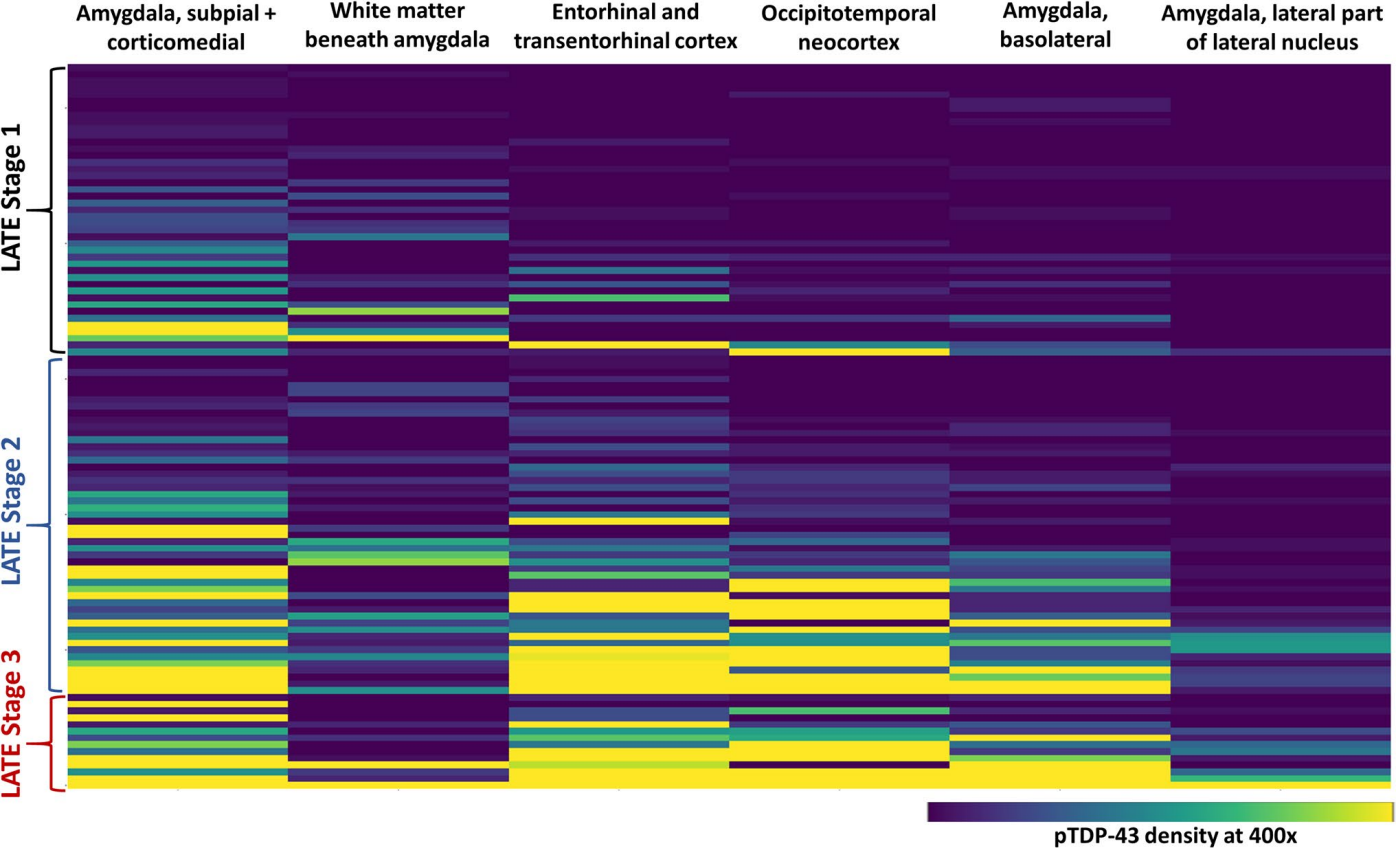
LATE-NC ordered by pTDP-43 severity and grouped by LATE-NC stage. This heat map shows the regional distribution of pTDP-43 pathology in six study ROIs in all subjects with LATE-NC, independent of inclusion morphology. Samples are grouped by LATE-NC stage 1 (top), stage 2 (middle) and stage 3 (bottom). Ordering of cases within each stage is based on the average pTDP-43 inclusion density across the ROIs, ordered from low to high. The density of pTDP-43 inclusion pathology is indicated by the bar at the bottom right of the heat map

Figure 4 shows a heatmap depicting the regional variation in pTDP-43 pathology, independent of morphologic type. In this heatmap, each ROI is a column (e.g., “white matter beneath amygdala”), each unique participant is a row, and samples are arranged by three LATE-NC stages (see labels at left). Within each LATE-NC stage, rows are arranged by the overall pathologic severity in the sample, such that the top rows within “LATE Stage 1” and “LATE Stage 2” are the samples with the mildest pTDP-43 pathology within that group.

In LATE-NC Stages 1 and 2, the ROIs with the earliest and mildest pathology were CM amygdala, WM, and ECtx/TECtx (Fig. 4). As TDP-43 pathologic burden increased within stages 1 and 2 LATE-NC, not only did CM, WM and ECtx/TECtx become more severely involved, but OTCtx and BL amygdala also became more involved. In contrast, the La-L region was rarely involved outside of more severe Stage 2 and Stage 3 samples. Thus, both within and across LATE-NC Stages, a pattern of medial to lateral and ventrolateral to dorsolateral anatomic involvement by TDP-43 pathology was seen.

As shown in Supplemental Fig. 9, there were additional regions with infrequent pTDP pathology. These included not only La-L, but also WM of the inferior longitudinal fasciculus (lateral to La-L), ventral extension of claustrum (lateral to La-L), the centromedian nuclear complex of the amygdala (abutting the entorhinal fissure), and the magnocellular neurons of basal forebrain.

## Discussion

This study builds on prior work to generate insights into the heterogeneity and evolution of LATE-NC in aged brains. As in earlier studies, LATE-NC was associated with HS, older age, cognitive impairment, and the *TMEM106B* risk genotype. Among amygdala region pathologies, LATE-NC was associated with myelin loss, entorhinal cortex cell dropout, astrocytosis, and white matter vascular pathology, as well as amygdala region ARTAG. Using a clustering algorithm, we found four different patterns of LATE-NC pathology, sometimes apparently initiated outside the “amygdala proper”, but within the amygdala region. Although TDP-43 pathologies converged on common anatomic foci at higher stages, the patterns identified were clinically, pathologically, and genetically differentiable. One of these patterns (Pattern 4, or δ) was commonly seen in persons dying without documented cognitive impairment, and in isolation may represent a subclinical pathology.

Methodological strengths of the study included that LATE-NC pathology was determined blinded to other pathologic, clinical, and genetic data. Participants included both TDP-43-negative samples and many brains in early stages of LATE-NC. The TDP-43 pathologic patterns were also determined using relatively unbiased clustering methods, and we correlated the pathologic findings with a large variety of additional clinical and neuropathologic data. Both phosphorylated and non-phosphorylated TDP-43 antibodies were used on serially sectioned brain portions, with the determination that either staining approach is suitable for the neuropathological evaluation of LATE-NC.

Earlier studies of TDP-43 pathology have identified stage 1 pathology within amygdala [28, 39, 42], with stage 2 being in hippocampus and higher stages involving neocortex, as well as subcortical structures. Notably, the “amygdala” is not a single structure, but rather a complex collection of disparate cell groups, bounded by disparate structures such as entorhinal cortex, basal forebrain, white matter tracts, and anterior hippocampus [2, 4, 33, 54]. When only one brain region was involved in the mildest forms of LATE-NC, the affected anatomical focus was often outside of the amygdala proper (e.g., subpial CM, white matter, or periallocortex). We, therefore, propose that the stage 1 of LATE-NC be referred to as “amygdala region”, rather than “amygdala”. This will harmonize the diagnostic criteria with observed phenomena and should increase the sensitivity of neuropathologic evaluation to detecting early LATE-NC. “Amygdala region” (LATE stage 1) would include cases with TDP-43 pathology limited to periallocortex (transentorhinal, entorhinal cortex), non-gray matter structures (subpial region overlying amygdala, white matter beneath amygdala), and even the anterior portion of hippocampus that abuts amygdala, often sampled in more caudal or obliquely oriented sections through amygdala. We note that just as LATE-NC stage 1 may be subclinical [42], there are analogous stages of other early pathologies (e.g., Braak NFT stages I–III, Thal Aβ phases 1–2) that also are often seen in cognitively normal persons [25, 45].

**Fig. 5:**
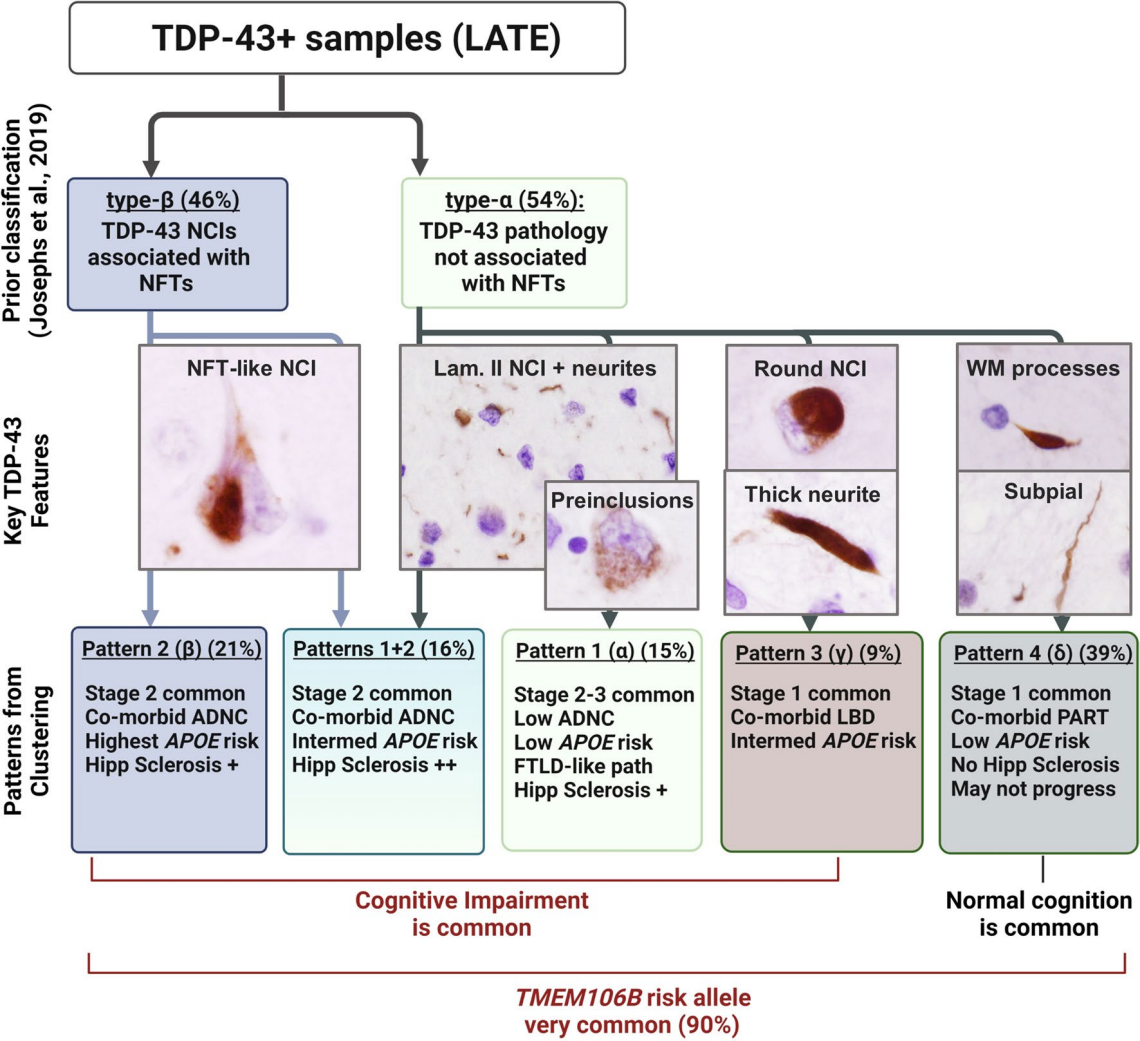
A proposed classification scheme for LATE-NC. Hierarchical clustering identified four patterns of LATE-NC (Patterns 1–4), and one group with combined features of Patterns 1 + 2, and the salient pathologic and clinical features are shown in this schematic. Consistent with the earlier work of Josephs et al. (2019), type-β is applied to the cluster-derived Pattern 2 (β). Also as in that earlier study, type-α refers to non-NFT associated TDP-43 pathology, but only to the FTLD-like lamina II pathology with frequent preinclusions (Pattern 1, or α). Schematic created (in part) with BioRender

A primary finding in the present study was that there were four patterns of LATE-NC, based on the clustering of 6 common types of TDP-43 pathologies. These patterns broadly replicate and extend an earlier landmark study, which identified two distinct patterns of LATE-NC, termed type-α and type-β [27]. A new proposed terminology reconciles both the prior work and the current study (Table 4), and this is further illustrated in the schematic of Fig. 5.

Analogous to the prior study by Josephs et al. [27], Pattern 1 (comparable to type α) refers specifically to those cases with FTLD-like lamina II pathology in periallocortical (TECtx, ECtx) and neocortical regions (OTCtx) of a section through amygdala. We also found that these cases have frequent granular preinclusions, readily detected by non-phosphorylated TDP-43 IHC, which may help to confirm the presence of this pattern in addition to its other features. Pattern 1 (type α) was associated with higher LATE-NC stages and non-NFT pathology. Pattern 2 (type β) with NFT-like TDP-43 NCIs was associated with tau-immunoreactive NFT pathology and lower LATE-NC stage. A difference from the prior study [27], which designated cases as either being type α or type β patterns, is that we also identified brains with coexisting features of both Pattern 1 (type-α) and Pattern 2 (type-β). This observation was previously described in a separate study wherein multiple neuropathologists assessed each case blindly [46]. Additional work is needed to determine whether the co-occurrence of TDP-43 morphologies in Pattern 1 + 2 cases is coincidental, or whether the different TDP-43 pathologies are synergistic with each other.

All TDP-43 pathologies studied here were identified using both phospho-TDP-43 and non-phospho-TDP-43 antibodies. The use of both antibodies is not required, although pTDP IHC was more sensitive to certain inclusion types (e.g., NFT-like, or type-β). Conversely, the non-phospho-TDP-43 antibody demonstrates both loss of nuclear labeling and early granular cytoplasmic inclusion pathology, and was more sensitive to granular preinclusions, as previously reported in ALS/FTLD-TDP spectrum brains [10, 13, 17]. Of note, granular preinclusions did not appear in all forms of advanced TDP-43 proteinopathy, and extensive Pattern 2 (type β) pathology with no granular preinclusion pathology could often be identified. In contrast, granular preinclusion pathology was a consistent feature in Pattern 1 (type-α) samples. The molecular factors contributing to this morphology deserve further investigation.

Brains with LATE-NC significantly differed from those without LATE-NC in several ways that were previously described, including increased frequencies of cognitive impairment [53] and comorbid HS [3, 26]. LATE-NC + samples also had more amygdala region ARTAG and myelin loss. Further, LATE-NC in all its forms was associated with the presence of a *TMEM106B* risk allele. This finding replicates earlier studies reporting an association between *TMEM106B* and LATE/HS-Aging [19, 27, 43]. The strong association between LATE-NC and *TMEM106B* in this study is notable provided the modest sample size, the widely varying severity and patterns of TDP-43 proteinopathy, and the inclusion of cognitively normal participants; thus, the *TMEM106B* risk allele is a key risk factor for LATE-NC across all patterns of pathology. Although the *APOE* risk allele did not necessarily associate with LATE-NC in Patterns 1 and 4, *APOE* ε4 was enriched in samples with prominent type-β NCI pathology (Pattern 2), and in samples with Pattern 3 (type γ).

Two novel patterns described here are proposed as Pattern 3 (type γ) and Pattern 4 (type δ). Pattern 3 samples had round NCIs and thick neurites in amygdala, usually without other forms of LATE-NC pathology. Pattern 3 was always associated with cognitive impairment and had the highest rate of transitional and diffuse LBD. Others have found that LATE-NC may have a unique phenotype in patients with LBD [1, 5, 52]. Although enriched in these cases, transitional and diffuse LBD was not exclusive to Pattern 3 (see Table 4), and other TDP-43 morphologies may emerge in higher LATE-NC stages in the setting of LBD.

Pattern 4 (type δ) was the most subtle subtype of LATE-NC, featuring TDP-43 + processes in subpial amygdala and/or subamygdaloid WM, often in the absence of NCI pathology. This corresponds to a morphology of TDP-43 first reported in detail by Arnold and colleagues [6], who also reported a similar anatomic distribution. While these TDP-43 immunoreactive structures may resemble neurites [34], in this study we demonstrate that these TDP-43 + processes were present in cells that were immunoreactive for CD44 (see Supplemental Fig. 7, online resource). CD44 is a glycoprotein expressed on the surface of the cell body and processes of astrocytes [21, 38]. By contrast, the TDP-43 + processes did not co-localize with three axonal markers. This suggests that the processes in Pattern 4 may be, at least in part, arising within astrocytes. The close association of Pattern 4 LATE-NC with corpora amylacea is also consistent with this interpretation.

Participants with Pattern 4 pathology in isolation had the lowest rates of ADNC and HS, and the lowest frequency of *APOE* ε4. Most Pattern 4 cases were LATE-NC Stage 1, and nearly half of the group was cognitively normal. Since some Pattern 4 cases were cognitively impaired, the pathological diagnosis is likely still warranted with the expectation that LATE-NC stage 1 may often be subclinical. However, our cross-sectional data, including many Pattern 4 cases with LATE-NC stage 1, raise the question as to whether some brains with Pattern 4 TDP-43 pathology in isolation will not progress further in terms of pathologic severity, perhaps analogous to how PART-may not progress to neocortical Braak NFT stages even in very advanced age [14]. An alternative hypothesis is that some Pattern 4 brains would have developed more pathology if they had progressed in age. One finding that suggests Pattern 4 may be more than a coincidental pathology is that these cases had an elevated frequency of the *TMEM106B* risk allele (90.5%), compared to samples without LATE-NC (74%). This does suggest a common pathogenetic pathway in the different LATE-NC subtypes, even with very mild pathology, and suggests that there may be compensatory mechanisms in some Pattern 4 cases. To test these hypotheses will require future studies.

There are limitations to the current work. The sample size was modest with a total of 184 cases studied. In addition, among study participants, the *APOE* risk allele rate (38.2% of study samples) indicated that there was a recruitment bias with a tendency for study samples to have higher-than-normal risk for developing ADNC. Other aspects of potential bias include the general tendency for brain banks to be enriched for high-socioeconomic status patients who may die at older ages than the general population and thus have elevated dementia risk. For definitive establishment of associations between LATE-NC patterns and clinical features, more work will be required. Future studies in larger cohorts, including more diverse samples from additional centers, are needed. An additional area not examined here and requiring further study is whether specific clinical syndromes were associated with LATE-NC patterns, or specific anatomic sites of involvement e.g., selective memory impairment, anomia, other language impairments, and/or prosopagnosia.

In summary, early foci of LATE-NC TDP-43 pathology occurred at several anatomic locations in the “amygdala region” and not only within the amygdala proper. Certain parameters and features, e.g., *APOE* risk alleles, HS, higher NFT stages, Lewy body pathology, and clinical impairment severity were associated with given patterns of LATE-NC. However, the *TMEM106B* risk allele was elevated in all LATE-NC patterns, which is remarkable given the heterogeneity of the TDP-43 pathology identified. The different TDP-43 patterns also tended to converge in the more severely affected brains, and each pattern could be seen at any given LATE-NC stage. Thus, our findings support the idea that there are meaningful commonalities under the umbrella diagnosis of LATE-NC.

## Supplementary Information

Below is the link to the electronic supplementary material.

Supplementary file1 (DOCX 25 KB)

Supplementary file2 (DOCX 97 KB)

Supplementary file3 Supplemental Figure 1. Scanned LFB/Nissl sections of two study samples, without (A) and with (B) atrophy in the amygdala region. The major study ROIs are shown, as also depicted in the schematic of Figure 1. (TIF 8410 KB)

Supplementary file4 Supplemental Figure 2. Microscopic heterogeneity within the amygdala region. LFB/Nissl images of representative fields as shown in the schematic at top center (all photomicrographs shown are at 200x magnification and the scale bar in the top right and lower right panels applies to all panels). Note the heterogeneity of cell density, cell orientation and cell size, even with components of “amygdala”, including ventral cortical nucleus (labeled “CM”), basal (BL) and accessory basal (AB) nuclei, lateral nucleus (Lat) and its lateral division (La-L). Because of the variability across cases, BL, AB, and Lat were grouped into a single ROI (“BL ROI”), and VCo and the parahippocampal-amygdaloid transition area (PHA) was grouped into a single ROI (“CM”). The central and medial nuclei were not incorporated into the CM nucleus because they were not consistent present across all samples; when present, these were never a significant site of early pTDP-43 pathology. (TIF 11119 KB)

Supplementary file5 Supplemental Figure 3. Subamygdaloid white matter and surrounding structures. H&E (panels A, C) and LFB/Nissl (panels B, D) stains show subamygdaloid white matter (“WM”), which is positioned between the basolateral division of amygdala (“Amygdala”) and entorhinal cortex (“ECtx”). This is a common site for conspicuous vascular pathologies, including venous collagenosis (panel C) and perivascular space widening (panels C, D). Corpora amylacea may also be abundant in this region (panel C). One of the morphologic measures in the amygdala region of this study was the combination of rarefied white matter (H&E) with loss of myelin (LFB), venous collagenosis, perivascular space widening and abundant corpora amylacea (scored as present or absent). The sample shown here demonstrates an example of the pattern being “present”. (TIF 11156 KB)

Supplementary file6 Supplemental Figure 4. Scoring of pTDP-43 inclusion density in amygdala region. pTDP-43 staining in four representative ROIs are shown in four different amygdalae with LATE-NC (one subject is shown per row). ROIs shown include basolateral nuclear group of amygdala (“BL”), the parahippocampal–amygdaloid transition zone of the CM ROI, layer 2 of the occipitotemporal cortex (“OTCtx”), and the subamygdaloid white matter (“WM”) that is positioned between amygdala and entorhinal cortex. The definitions of scores 0–4 are further defined in the Methods and considered all study ROIs, not just those depicted here. (TIF 8320 KB)

Supplementary file7 Supplemental Figure 5. Additional pathologic measures in the amygdala region. Pathologies assessed in the amygdala region, blinded to pTDP-43 status, included fibrillary astrocytosis (H&E) (top row), assessed as present/absent, as well as three-tiered scales to assess neuronal loss in entorhinal cortex (Nissl/LFB) (middle row) and myelin loss (bottom row), particularly in white matter between amygdala and entorhinal cortex. Top row images are from the deep laminae of entorhinal cortex and conspicuous reactive astrocytes are indicated by white arrows. Middle row images are centered on the layer V (pri-α) of entorhinal cortex, and this layer as well as the overlying lamina dissecans (“Lam. Diss”) are labeled. Scale bars for right-most images apply to all images on that row. (TIF 10386 KB)

Supplementary file8 Supplemental Figure 6. Overlapping and non-overlapping TDP-43 and Tau pathologies in LATE-NC. (A) The short non-tapering neurites in lamina II that characterized Pattern 1 (type-α) did not co-localize with tau-positive pathology. (B) In contrast, TDP-43 and Tau co-localized in the same neurons and even cytoplasmic foci in this example of a Pattern 2 (type-β). (C) LATE-NC was significantly associated with amygdala region ARTAG, although the TDP-43 processes of Pattern 4 and tau-positive ARTAG did not co-localize. (TIF 6085 KB)

Supplementary file9 Supplemental Figure 7. CD44 and TDP-43 labeling in WM and subpial amygdala. CD44 is a non-specific marker, that can be used to highlight astrocytic membranes, as shown in the control CD44-GFAP image in panel A. In WM (B, C, and D), CD44 staining and TDP-43 stains show the proximity of TDP-43+ processes and CD44+ membranes, suggesting some of this TDP-43 pathology may occur within astrocytes. Note that in panel D (subpial region of amygdala), TDP-43+ process wrap around corpora amylacea, appearing as a negative space in the image surrounded by CD44+ processes. Panel C is enlarged from the region-of-interest in panel B. Panels D and E are enlarged, and a scale bar is provided for both. (TIF 10262 KB)

Supplementary file10 Supplemental Figure 8. TDP-43-positive processes and axonal markers. Select samples with Pattern 4 (δ), characterized by TDP-43+ processes, were also investigated using double labeling for TDP-43 and three neuronal and axonal markers. As shown in WM (A) and subpial amygdala (B), no overlap was seen. The possibility that some of this pathology is neuritic/axonal cannot entirely be excluded, but better co-localization was seen in foci of dense CD44 staining (Supplemental Fig. 7) and GFAP labeling (not shown). Scale bar applies to both images. (TIF 8378 KB)

Supplementary file11 Supplemental Figure 9. Regions resistant to pTDP-43 pathology in LATE-NC. Paired LFB/Nissl and pTDP-43 stains in ROIs in a single study subject with mild LATE-NC in the amygdala region. Three ROIs frequently positive in LATE-NC are highlighted with a red outline at the bottom right (images taken at 600x and the scale bar for “Amygdala, BL” applies to all three sets of ROIs). In contrast, negative ROIs dorsally and laterally are shown, including ventrolateral extent of basal forebrain, ventral extension of claustrum (near La-L), the lateral subdivision of the lateral nucleus of amygdala (La-L), the inferior longitudinal fasciculus (ILF) (in contrast, subamygdaloid WM was positive in this subject), and centromedian nuclear complex. This subject is representative of many cases with this medial to ventrolateral and lateral gradient of pTDP-43 pathology in LATE-NC. (TIF 8882 KB)

## Data Availability

Data and/or supporting microscopic images from this study are available from the corresponding author upon reasonable request.
