# Supplementary material for: Patterns of amygdala region pathology in LATE-NC: subtypes that differ with regard to TDP-43 histopathology, genetic risk factors, and comorbid pathologies
Source: Acta Neuropathol. 2022 Apr 2;143(5):531–45. doi: 10.1007/s00401-022-02416-5 (PMC9038848; doi:10.1007/s00401-022-02416-5)
Supplement: Supplementary file 1 — Supplementary file1 (DOCX 25 KB) [file 401_2022_2416_MOESM1_ESM.docx]

**Supplemental File - Materials and Methods**

**TDP-43, pTDP, and other antibodies**

pTDP and TDP-43 IHC were used on all blocks (see main manuscript file). The non-phosphorylation-dependent antibody recognizes all post-translationally modified and truncated forms of TDP-43, as well as full-length physiologic TDP-43 (Proteintech, catalogue ID 10782-2-AP, rabbit polyclonal, 1:1000, Rosemont, IL, USA). The adjacent section per FFPE block was stained for phospho-TDP43 (Proteintech, catalogue ID 22309-1-AP, rabbit polyclonal, 1:500, Rosemont, IL, USA), which recognizes TDP-43 phosphorylated at serine residues at 409 and 410.

**Immunostaining procedures**

Deparaffinization and rehydration steps were carried out on 6 µm FFPE sections, using sequential washes in reagent grade xylene, graded alcohols, and water. Heat-based antigen retrieval was performed using an antigen retrieval solution at pH 9 (Agilent Technologies; Santa Clara, CA) for 1 hour (30 min at 95° C followed by 30 min on ice). Washing steps were carried out using a commercial Tris-buffered saline solution with Tween 20, pH 7.6 (Agilent Technologies). Endogenous peroxide was blocked using a 3% hydrogen peroxide solution (VWR International; Radnor, PA). Following a 1-hour blocking step at room temperature (2.5% horse serum, Vector Laboratories, Burlingame, CA), primary antibody was applied overnight at 4°C. Slides were thoroughly washed and the ImmPress horseradish peroxidase (HRP) anti-rabbit and anti-mouse IgG detection kits (Vector Laboratories) were applied as appropriate for 1-hour at room temperature. Target antigen was visualized using DAB chromogen in substrate buffer (Agilent Technologies) with timing established by two study authors (MDC, ASA) from control autopsy material. Hematoxylin counterstain was applied after additional washing steps and slides were brought to xylene and mounted with Permount (ThermoFisher Scientific; Waltham, MA).

**Immunofluorescence studies**

Antibodies for double labeling included mouse monoclonal Tau (AT8) and rabbit polyclonal TDP-43 (details above), as well as an additional TDP-43 antibody (Proteintech, catalogue ID 66079-1-IG, mouse monoclonal, 1:100, Rosemont, Illinois, USA), MAP-2 (Abcam, catalogue ID ab32454, rabbit polyclonal, 1:100, Cambridge, MA, USA), Tubulin (EMD Millipore, catalogue ID 05-559-I, mouse monoclonal, 1:100, Burlington, MA, USA), pan-neuronal marker (neurofilament cocktail) (BioLegend, catalogue ID SMI-311R, mouse monoclonal, 1:100, San Diego, CA, USA), GFAP (Abcam, catalogue ID ab7260, rabbit polyclonal, 1:250, Cambridge, MA, USA) and CD44 (R&D Systems, catalogue ID BBA10, mouse monoclonal, 1:50, Minneapolis, MN, USA).

Briefly, slides followed the deparaffinization, rehydration, antigen retrieval, and blocking steps of IHC described above with all washing steps using fresh PBS. The mixture of primary antibodies was incubated on the FFPE section overnight at 4°C and the mixture of secondary antibodies were applied for 1 hour at room temperature (Alexa Fluor 555 Anti-Rabbit IgG (1:200; A21429) and Alexa Fluor 488 anti-Mouse IgG (1:200; A11001) (Alexa Fluor are products of ThermoFisher). After additional PBS washing steps, slides were mounted using

Vectashield Antifade mounting medium with 4′,6-diamidino-2-phenylindole (DAPI; Vector Laboratories). Slides were examined on an Olympus BX-43 Microscope housing an enhanced

green fluorescent protein (EGFP) FITC/Cy2 filter cube (set number 49002, Olympus; Center Valley, PA), a CY3/tetramethylrhodamine-isothiocyanate (TRITC) filter cube (set number 49004, Olympus), a DAPI filter cube (set number 49000, Olympus), and a triple-band filter set (ET D/F/R 69000, Olympus). Images of DAPI, FITC, and TRITC filters were captured separately with an Olympus DP71 Camera and merged in cellSens software 1.13 (Olympus America, Inc.; Center Valley, PA).

**Single nucleotide polymorphisms (SNP) genotyping**

Isolated gDNA sample purity was checked in a Nanodrop 2000 spectrophotometer (Thermo Fisher Scientific, USA) and gDNA samples with a 260/280 ratio > 1.8 were used for genotyping. SNP genotyping was performed using TaqMan^TM^ probes for the following SNPs: *APOE* rs429358/ rs7412, *ABCC9* rs704180, *GRN* rs5848, *KCNMB2* rs12496790, and *TMEM106B* rs1990622 (Thermo Fisher Scientific, USA). Each PCR reaction mixture contained 1x TaqMan^TM^ Genotyping Master Mix (catalogue ID 4371355, Applied Biosystems, USA), 0.25 μM of TaqMan^TM^ probe, and 20 ng of gDNA. A no template control with nuclease-free water was performed with each sequencing run. PCR amplification protocol was as follows: initial activation of AmpliTaq Gold® DNA Polymerase at 95°C for 10 min, followed by 40 cycles with denaturation at 95 °C for 20 sec, annealing at 60°C for 1.30 mins, followed by extension at 72°C for 30 sec. The amplification was performed using the Bio-Rad’s CFX 96 Real Time System (Bio-Rad, USA). Interpretation of SNP data was performed using the Allelic Discrimination function in CFX Maestro Software (Bio-Rad, USA).
