# Supplementary material for: Patterns of amygdala region pathology in LATE-NC: subtypes that differ with regard to TDP-43 histopathology, genetic risk factors, and comorbid pathologies
Source: Acta Neuropathol. 2022 Apr 2;143(5):531–45. doi: 10.1007/s00401-022-02416-5 (PMC9038848; doi:10.1007/s00401-022-02416-5)
Supplement: Supplementary file 2 — Supplementary file2 (DOCX 97 KB) [file 401_2022_2416_MOESM2_ESM.docx]

**Supplemental Table 1. Pattern, pTDP severity, semiquantitative (pTDP), qualitative (pTDP+TDP43 IHC), Braak stage (NFT), Cognitive, Interval, Hipp Scl (page 1/6)**

| **ID** | **Cluster** | **Group** | **pTDP** | **OTCtx** | **TECtx**  **ECTx** | **CM** | **BL** | **LaL** | **WM** | **Conspic. ROI** | **β** | **Lam II** | **Round NCI** | **WM** | **Subpial** | **Gran**  **Preinc** | **Thick Neur** | **NFT** | **Cog** | **Int** | **HS** |
| --- | --- | --- | --- | --- | --- | --- | --- | --- | --- | --- | --- | --- | --- | --- | --- | --- | --- | --- | --- | --- | --- |
| **T042** | Pattern 1 | Alpha | 4 | 30 | 30 | 30 | 30 | 20 | 3 | Amygdala | 0 | 1 | 0 | 1 | 0 | 1 | 0 | 5 | 2 | 0.3 | 1 |
| **T061** | Pattern 1 | Alpha | 3 | 30 | 30 | 11 | 5 | 12 | 0 | TECtx | 0 | 1 | 1 | 0 | 1 | 1 | 0 | 3 | 1 | 0.5 | 0 |
| **T115** | Pattern 1 | Alpha | 3 | 21 | 7 | 2 | 3 | 1 | 0 | Occipitotemporal Ctx | 0 | 1 | 0 | 0 | 1 | 1 | 0 | 2 | 2 | IA | 1 |
| **T129** | Pattern 1 | Alpha | 2 | 1 | 8 | 0 | 4 | 0 | 4 | ERCtx | 0 | 1 | 0 | 1 | 0 | 1 | 0 | 4 | 0 | IA | 0 |
| **T140** | Pattern 1 | Alpha | 4 | 18 | 22 | 7 | 30 | 2 | 4 | Amygdala | 0 | 1 | 1 | 1 | 1 | 1 | 0 | 4 | 2 | IA | 1 |
| **T146** | Pattern 1 | Alpha | 2 | 5 | 3 | 4 | 2 | 0 | 4 | White Matter, Occipitotemporal Ctx | 0 | 1 | 0 | 1 | 1 | 1 | 0 | 3 | 0 | 0.6 | 0 |
| **T148** | Pattern 1 | Alpha | 1 | 5 | 12 | 15 | 0 | 0 | 0 | ERCtx | 0 | 1 | 0 | 0 | 1 | 1 | 0 | 2 | 0 | 0.2 | 0 |
| **T150** | Pattern 1 | Alpha | 3 | 1 | 30 | 30 | 3 | 0 | 7 | TECtx | 0 | 1 | 0 | 1 | 1 | 1 | 0 | 1 | 0 | IA | 0 |
| **T151** | Pattern 1 | Alpha | 3 | 8 | 30 | 30 | 30 | 5 | 3 | Amygdala | 0 | 1 | 1 | 1 | 1 | 1 | 0 | 0 | 0 | 0.6 | 0 |
| **T152** | Pattern 1 | Alpha | 4 | 30 | 30 | 30 | 30 | 2 | 15 | Occipitotemporal ctx, TECtx | 0 | 1 | 0 | 1 | 1 | 1 | 0 | 2 | 0 | 1.0 | 0 |
| **T157** | Pattern 1 | Alpha | 4 | 30 | 30 | 30 | 30 | 6 | 2 | Occipitotemporal Ctx | 0 | 1 | 0 | 1 | 1 | 1 | 0 | 2 | 1 | 1.0 | 1 |
| **T164** | Pattern 1 | Alpha | 4 | 30 | 30 | 30 | 24 | 2 | 1 | Occipitotemporal Ctx | 0 | 1 | 1 | 1 | 1 | 1 | 0 | 3 | 2 | IA | 1 |
| **T165** | Pattern 1 | Alpha | 3 | NaN | 30 | 30 | 30 | NaN | 30 | Amygdala | 0 | 1 | 0 | 1 | 1 | 1 | 0 | 1 | 1 | 0.5 | 1 |
| **T169** | Pattern 1 | Alpha | 4 | 30 | 30 | 8 | 7 | NaN | 5 | Occipitotemporal ctx | 1 | 1 | 0 | 1 | 1 | 1 | 0 | 5 | 1 | 2.5 | 1 |
| **T040** | Pattern 1 | Alpha | 1 | 0 | 1 | 7 | 1 | 0 | 0 | Amygdala | 1 | 0 | 1 | 0 | 1 | 1 | 0 | 6 | 2 | 0.6 | 1 |
| **T182** | Pattern 1 | Alpha | 2 | 3 | 15 | 0 | 8 | 0 | 25 | TECtx | 1 | 0 | 0 | 1 | 0 | 1 | 0 | 5 | 1 | 2.4 | 1 |
| **T012** | Pattern 2 | Beta | 2 | 1 | 21 | 1 | 1 | 0 | 0 | TECtx/ERCtx | 1 | 0 | 1 | 0 | 0 | 0 | 0 | 5 | 2 | 0.0 | 1 |
| **T018** | Pattern 2 | Beta | 2 | 1 | 6 | 1 | 1 | 0 | 0 | TECtx | 1 | 0 | 1 | 0 | 0 | 0 | 0 | 5 | 2 | 1.0 | 1 |
| **T020** | Pattern 2 | Beta | 3 | 14 | 30 | 3 | 7 | 0 | 0 | TECTx | 1 | 0 | 1 | 0 | 0 | 0 | 0 | 6 | 2 | 0.1 | 0 |
| **T025** | Pattern 2 | Beta | 2 | 2 | 7 | 2 | 1 | 0 | 0 | TECtx | 1 | 0 | 1 | 0 | 0 | 0 | 0 | 5 | 2 | 2.5 | 1 |
| **T027** | Pattern 2 | Beta | 2 | 1 | 12 | 15 | 2 | 1 | 11 | ERCtx | 1 | 0 | 1 | 1 | 1 | 0 | 0 | 5 | 2 | 2.2 | 1 |
| **T030** | Pattern 2 | Beta | 2 | 2 | 7 | 12 | 2 | 1 | 0 | TECtx, Amygdala | 1 | 0 | 1 | 0 | 0 | 0 | 0 | 6 | 2 | 0.8 | 1 |
| **T032** | Pattern 2 | Beta | 3 | 0 | 12 | 30 | 30 | 2 | 6 | Amygdala | 1 | 0 | 1 | 1 | 0 | 0 | 0 | 6 | 2 | 0.6 | 1 |
| **T034** | Pattern 2 | Beta | 2 | 12 | 5 | 30 | 6 | 1 | 0 | Amygdala | 1 | 0 | 1 | 0 | 0 | 0 | 0 | 6 | 2 | 3.5 | 1 |
| **T038** | Pattern 2 | Beta | 1 | NaN | 1 | 0 | 0 | 0 | 0 | ERCtx | 1 | 0 | 0 | 0 | 0 | 0 | 0 | 2 | 0 | 1.5 | 0 |
| **T046** | Pattern 2 | Beta | 1 | 0 | 1 | 3 | 1 | 0 | 4 | Amygdala | 1 | 0 | 1 | 1 | 0 | 0 | 0 | 3 | 0 | 0.9 | 0 |
| **T049** | Pattern 2 | Beta | 1 | 2 | 3 | 4 | 2 | 0 | 2 | Amygdala | 1 | 0 | 1 | 1 | 0 | 0 | 1 | 6 | 2 | 0.0 | 0 |
| **T058** | Pattern 2 | Beta | 1 | 0 | 2 | 0 | 0 | 0 | 0 | TECtx | 1 | 0 | 0 | 0 | 0 | 0 | 0 | 6 | 2 | 0.5 | 0 |
| **T059** | Pattern 2 | Beta | 3 | 30 | 30 | 24 | 13 | 1 | 4 | Amygdala | 1 | 0 | 1 | 1 | 1 | 0 | 0 | 5 | 2 | 0.1 | 1 |
| **T060** | Pattern 2 | Beta | 2 | 1 | 11 | 1 | 2 | 1 | 0 | ERCtx | 1 | 0 | 1 | 0 | 0 | 0 | 0 | 6 | 2 | 0.4 | 0 |
| **T063** | Pattern 2 | Beta | 2 | 2 | 4 | 1 | 3 | 1 | 0 | Amygdala | 1 | 0 | 1 | 0 | 0 | 0 | 0 | 5 | 2 | 0.2 | 1 |
| **T107** | Pattern 2 | Beta | 1 | 0 | 3 | 0 | 0 | 0 | 0 | ERCtx | 1 | 0 | 0 | 0 | 0 | 0 | 0 | 5 | 2 | IA | 0 |
| **T130** | Pattern 2 | Beta | 3 | 0 | 27 | 30 | 30 | 0 | 30 | Amygdala | 1 | 0 | 1 | 1 | 1 | 0 | 1 | 6 | 2 | IA | 0 |
| **T143** | Pattern 2 | Beta | 1 | NaN | 1 | 0 | 0 | 0 | 0 | ERCtx | 1 | 0 | 0 | 0 | 0 | 0 | 0 | 4 | 0 | 0.6 | 0 |
| **T153** | Pattern 2 | Beta | 2 | NaN | 3 | 1 | 3 | 0 | 0 | ERCtx | 1 | 0 | 0 | 0 | 0 | 0 | 0 | 3 | 0 | 2.0 | 1 |
| **T163** | Pattern 2 | Beta | 2 | NaN | 10 | 0 | 0 | NaN | 0 | TECtx/ECtx | 1 | 0 | 0 | 0 | 0 | 0 | 0 | 6 | 1 | 0.4 | 0 |
| **T171** | Pattern 2 | Beta | 3 | 15 | 10 | 30 | 22 | NaN | 2 | Amygdala | 1 | 0 | 1 | 1 | 1 | 0 | 0 | 5 | 1 | 0.5 | 1 |
| **T178** | Pattern 2 | Beta | 1 | 0 | 0 | 2 | 0 | 0 | 0 | Amygdala | 1 | 0 | 0 | 0 | 0 | 0 | 0 | 6 | 2 | 2.5 | 1 |
| **T001** | Combo 1+2 | Beta+  Alpha | 3 | 30 | 3 | 14 | 21 | 0 | 0 | Occipitotemporal ctx | 1 | 1 | 1 | 0 | 1 | 0 | 0 | 5 | 2 | 2.2 | 1 |
| **T002** | Combo 1+2 | Beta+  Alpha | 3 | 30 | 8 | 9 | 9 | 1 | 17 | Occipitotemporal ctx | 1 | 1 | 1 | 1 | 0 | 1 | 0 | 5 | 2 | IA | 1 |
| **T005** | Combo 1+2 | Beta+  Alpha | 3 | 30 | 29 | 14 | 7 | 3 | 14 | TECtx/ERCtx | 1 | 1 | 1 | 1 | 1 | 0 | 0 | 4 | 2 | 0.9 | 1 |
| **T028** | Combo 1+2 | Beta+  Alpha | 4 | 30 | 30 | 30 | 23 | 6 | 0 | Occipitotemporal Ctx, Amygdala | 1 | 1 | 1 | 0 | 0 | 0 | 0 | 6 | 2 | 1.8 | 1 |
| **T031** | Combo 1+2 | Beta+  Alpha | 3 | 30 | 12 | 11 | 7 | 6 | 15 | Occipitotemporal Ctx | 1 | 1 | 1 | 1 | 1 | 0 | 0 | 4 | 2 | 0.2 | 1 |
| **T036** | Combo 1+2 | Beta+  Alpha | 4 | 30 | 30 | 15 | 30 | 10 | 5 | Occipitotemporal Ctx, Amygdala, TECtx | 1 | 1 | 1 | 1 | 1 | 0 | 0 | 5 | 2 | 2.8 | 1 |
| **T041** | Combo 1+2 | Beta+  Alpha | 2 | 10 | 7 | 3 | 0 | 1 | 18 | TECtx/ERCtx | 1 | 1 | 1 | 1 | 1 | 0 | 0 | 5 | 2 | 0.0 | 1 |
| **T048** | Combo 1+2 | Beta+  Alpha | 2 | 30 | 2 | 14 | 9 | 4 | 3 | Occipitotemporal Ctx, Amygdala | 1 | 1 | 1 | 1 | 1 | 0 | 0 | 5 | 2 | 0.3 | 0 |
| **T092** | Combo 1+2 | Beta+  Alpha | 3 | 30 | 30 | 6 | 3 | 3 | 2 | Occipitotemporal ctx | 1 | 1 | 1 | 1 | 0 | 1 | 0 | 5 | 2 | IA | 1 |
| **T112** | Combo 1+2 | Beta+  Alpha | 3 | NaN | 30 | 15 | 12 | NaN | 3 | ERCtx, Amygdala | 1 | 1 | 1 | 1 | 1 | 0 | 0 | 5 | 2 | IA | 1 |
| **T166** | Combo 1+2 | Beta+  Alpha | 2 | 5 | 30 | 2 | 8 | 0 | 3 | ERCtx | 1 | 1 | 1 | 1 | 1 | 1 | 0 | 3 | 1 | 5.3 | 1 |
| **T167** | Combo 1+2 | Beta+  Alpha | 2 | 1 | 30 | 1 | 2 | 0 | 0 | TECtx | 1 | 1 | 1 | 0 | 0 | 0 | 0 | 3 | 1 | 1.6 | 1 |
| **T083** | Combo 1+2 | Beta+  Alpha | 3 | 30 | 12 | 24 | 11 | 10 | 0 | Amygdala, Occipitotemporal Ctx | 1 | 1 | 1 | 0 | 1 | 1 | 0 | 4 | 2 | IA | 1 |
| **T090** | Combo 1+2 | Beta+  Alpha | 3 | 5 | 22 | 30 | 5 | 1 | 0 | Amygdala | 1 | 1 | 1 | 0 | 0 | 1 | 0 | 5 | 2 | IA | 1 |
| **T141** | Combo 1+2 | Beta+  Alpha | 3 | 30 | 30 | 10 | 3 | 0 | 0 | Occipitotemporal ctx | 1 | 1 | 1 | 0 | 1 | 1 | 0 | 6 | 2 | IA | 0 |
| **T142** | Combo 1+2 | Beta+  Alpha | 3 | 30 | 3 | 24 | 12 | 1 | 0 | Occipitotemporal ctx | 1 | 1 | 1 | 0 | 1 | 1 | 0 | 6 | 2 | IA | 0 |
| **T175** | Combo 1+2 | Beta+  Alpha | 2 | 5 | 7 | 2 | 2 | 1 | 0 | ERCtx | 1 | 1 | 1 | 0 | 1 | 1 | 0 | 3 | 2 | 2.2 | 1 |
| **T068** | Pattern 4 | Delta | 1 | 0 | 0 | 30 | 2 | 0 | 4 | Subpial | 0 | 0 | 1 | 1 | 1 | 0 | 0 | 1 | 2 | 0.4 | 0 |
| **T156** | Pattern 4 | Delta | 2 | 5 | 5 | 5 | 12 | 0 | 22 | White Matter | 0 | 1 | 0 | 1 | 0 | 0 | 0 | 2 | 0 | 0.9 | 0 |
| **T159** | Pattern 4 | Delta | 2 | NaN | 7 | 3 | 6 | 0 | 1 | TECtx | 0 | 1 | 0 | 1 | 1 | 0 | 0 | 2 | 0 | 1.5 | 1 |
| **T168** | Pattern 4 | Delta | 1 | 0 | 2 | 0 | 0 | 0 | 6 | White Matter | 0 | 0 | 0 | 1 | 0 | 0 | 0 | 2 | 1 | 2.2 | 1 |
| **T009** | Pattern 4 | Delta | 1 | 0 | 0 | 1 | 0 | 0 | 0 | Subpial | 0 | 0 | 0 | 0 | 1 | 0 | 0 | 2 | 0 | 0.9 | 0 |
| **T011** | Pattern 4 | Delta | 1 | 0 | 0 | 1 | 0 | 0 | 0 | Subpial | 0 | 0 | 0 | 0 | 1 | 0 | 0 | 5 | 2 | 0.2 | 0 |
| **T015** | Pattern 4 | Delta | 1 | 0 | 0 | 0 | 0 | 0 | 3 | White Matter | 0 | 0 | 0 | 1 | 0 | 0 | 0 | 1 | 2 | 0.3 | 0 |
| **T016** | Pattern 4 | Delta | 1 | 0 | 0 | 16 | 0 | 0 | 0 | Subpial | 0 | 0 | 0 | 0 | 1 | 0 | 0 | 1 | 0 | 0.3 | 0 |
| **T021** | Pattern 4 | Delta | 1 | 0 | 0 | 2 | 0 | 0 | 0 | Subpial | 0 | 0 | 0 | 0 | 1 | 0 | 0 | 0 | 0 | 1.2 | 0 |
| **T022** | Pattern 4 | Delta | 1 | 0 | 0 | 1 | 0 | 0 | 2 | White Matter | 0 | 0 | 0 | 1 | 1 | 0 | 0 | 3 | 1 | 1.3 | 0 |
| **T026** | Pattern 4 | Delta | 1 | 0 | 0 | 1 | 1 | 0 | 0 | Amygdala | 0 | 0 | 1 | 0 | 1 | 0 | 0 | 5 | 1 | 1.0 | 0 |
| **T047** | Pattern 4 | Delta | 1 | 0 | 0 | 1 | 0 | 0 | 1 | Subpial, White Matter | 0 | 0 | 0 | 1 | 1 | 0 | 0 | 1 | 0 | 1.0 | 0 |
| **T056** | Pattern 4 | Delta | 1 | 0 | 0 | 1 | 0 | 0 | 12 | White Matter | 0 | 0 | 0 | 1 | 1 | 0 | 0 | 6 | 2 | 1.3 | 0 |
| **T057** | Pattern 4 | Delta | 1 | 0 | 0 | 0 | 0 | 0 | 25 | White Matter | 0 | 0 | 0 | 1 | 0 | 0 | 0 | 2 | 0 | 3.1 | 0 |
| **T064** | Pattern 4 | Delta | 1 | NaN | 0 | 16 | 0 | 0 | 0 | Subpial | 0 | 0 | 0 | 0 | 1 | 0 | 0 | 5 | 2 | 0.6 | 0 |
| **T066** | Pattern 4 | Delta | 1 | 0 | 0 | 23 | 0 | 0 | 30 | White Matter | 0 | 0 | 0 | 1 | 1 | 0 | 0 | 1 | 1 | 5.1 | 0 |
| **T071** | Pattern 4 | Delta | 1 | 0 | 0 | 0 | 0 | 0 | 1 | White Matter | 0 | 0 | 0 | 1 | 0 | 0 | 0 | 4 | 2 | 0.3 | 0 |
| **T073** | Pattern 4 | Delta | 1 | 0 | 0 | 9 | 0 | 0 | 0 | Subpial | 0 | 0 | 0 | 0 | 1 | 0 | 0 | 1 | 1 | 1.0 | 0 |
| **T078** | Pattern 4 | Delta | 1 | NaN | 0 | 0 | 0 | 0 | 7 | White Matter | 0 | 0 | 0 | 1 | 0 | 0 | 0 | 1 | 2 | IA | 0 |
| **T082** | Pattern 4 | Delta | 1 | 0 | 0 | 6 | 0 | 0 | 5 | Subpial | 0 | 0 | 0 | 1 | 1 | 0 | 0 | 4 | NaN | IA | 0 |
| **T087** | Pattern 4 | Delta | 1 | 0 | 0 | 8 | 0 | 0 | 0 | Subpial | 0 | 0 | 0 | 0 | 1 | 0 | 0 | 3 | 1 | IA | 0 |
| **T091** | Pattern 4 | Delta | 1 | 0 | 0 | 0 | 0 | 0 | 6 | White Matter | 0 | 0 | 0 | 1 | 0 | 0 | 0 | 4 | 0 | IA | 0 |
| **T093** | Pattern 4 | Delta | 1 | NaN | 0 | 4 | 0 | 0 | 0 | Subpial | 0 | 0 | 0 | 0 | 1 | 0 | 0 | 4 | 0 | IA | 0 |
| **T096** | Pattern 4 | Delta | 1 | 0 | 0 | 15 | 0 | 0 | 2 | Subpial | 0 | 0 | 0 | 1 | 1 | 0 | 0 | 1 | 1 | IA | 0 |
| **T109** | Pattern 4 | Delta | 1 | 0 | 0 | 0 | 0 | 0 | 5 | White Matter | 0 | 0 | 0 | 1 | 0 | 0 | 0 | 2 | 0 | IA | 0 |
| **T110** | Pattern 4 | Delta | 1 | 0 | 0 | 14 | 0 | 0 | 0 | Subpial | 0 | 0 | 0 | 0 | 1 | 0 | 0 | 3 | 2 | IA | 0 |
| **T113** | Pattern 4 | Delta | 1 | 0 | 0 | 18 | 0 | 0 | 7 | Subpial, White Matter | 0 | 0 | 0 | 1 | 1 | 0 | 0 | 2 | 2 | IA | 0 |
| **T144** | Pattern 4 | Delta | 1 | NaN | 0 | 19 | 0 | 0 | 2 | Subpial | 0 | 0 | 0 | 1 | 1 | 0 | 0 | 2 | 0 | 0.7 | 0 |
| **T145** | Pattern 4 | Delta | 2 | NaN | 0 | 30 | 0 | 0 | 0 | Subpial | 0 | 0 | 0 | 0 | 1 | 0 | 0 | 4 | 0 | 0.5 | 0 |
| **T147** | Pattern 4 | Delta | 1 | 0 | 0 | 3 | 0 | 0 | 5 | Subpial, White Matter | 0 | 0 | 0 | 1 | 1 | 0 | 0 | 3 | 0 | 0.9 | 0 |
| **T149** | Pattern 4 | Delta | 1 | 0 | 0 | 12 | 0 | 0 | 0 | Subpial | 0 | 0 | 0 | 0 | 1 | 0 | 0 | 1 | 0 | IA | 0 |
| **T154** | Pattern 4 | Delta | 1 | 0 | 5 | 2 | 3 | 0 | 0 | ERCtx | 0 | 0 | 0 | 0 | 1 | 0 | 0 | 0 | 0 | 1.0 | 0 |
| **T155** | Pattern 4 | Delta | 1 | NaN | 0 | 18 | 0 | 0 | 2 | Subpial | 0 | 0 | 0 | 1 | 1 | 0 | 0 | 3 | 1 | 0.1 | 0 |
| **T158** | Pattern 4 | Delta | 2 | 0 | 7 | 30 | 0 | 0 | 0 | Subpial | 0 | 0 | 0 | 0 | 1 | 0 | 0 | 1 | 0 | 1.5 | 1 |
| **T160** | Pattern 4 | Delta | 1 | 0 | 0 | 10 | 0 | 0 | 5 | Subpial | 0 | 0 | 0 | 1 | 1 | 0 | 0 | 1 | 0 | 0.8 | 0 |
| **T162** | Pattern 4 | Delta | 1 | 0 | 0 | 30 | 0 | 0 | 0 | CM/subpial processes | 0 | 0 | 0 | 0 | 1 | 0 | 0 | 5 | 0 | 0.9 | 1 |
| **T170** | Pattern 4 | Delta | 1 | 0 | 0 | 0 | 0 | 0 | 6 | White Matter | 0 | 0 | 0 | 1 | 0 | 0 | 0 | 2 | 2 | 0.2 | 0 |
| **T176** | Pattern 4 | Delta | 2 | 0 | 0 | 30 | 0 | 0 | 5 | Subpial | 0 | 0 | 0 | 1 | 1 | 0 | 0 | 3 | 1 | 0.2 | 1 |
| **T177** | Pattern 4 | Delta | 2 | 0 | 0 | 30 | 0 | 0 | 15 | Subpial | 0 | 0 | 0 | 1 | 1 | 0 | 0 | 3 | 2 | 1.9 | 0 |
| **T181** | Pattern 4 | Delta | 1 | NaN | NaN | 9 | 0 | 0 | 0 | Subpial | 0 | 0 | 0 | 0 | 1 | 0 | 0 | 4 | 2 | IA | 0 |
| **T183** | Pattern 4 | Delta | 2 | 0 | 0 | 7 | 0 | 0 | 4 | Subpial | 0 | 0 | 0 | 1 | 0 | 0 | 0 | 4 | 2 | 2.8 | 1 |
| **T184** | Pattern 4 | Delta | 1 | 0 | 5 | 2 | 0 | 0 | 0 | Subpial | 0 | 0 | 0 | 0 | 1 | 0 | 0 | 2 | 1 | 1.9 | 0 |
| **T017** | Pattern 3 | Gamma | 2 | 0 | 0 | 3 | 1 | 1 | 0 | Amygdala | 0 | 0 | 1 | 0 | 0 | 0 | 1 | 5 | 2 | 0.2 | 0 |
| **T029** | Pattern 3 | Gamma | 3 | 17 | 16 | 18 | 5 | 7 | 0 | Occipitotemporal ctx, TECtx | 0 | 1 | 1 | 0 | 0 | 1 | 1 | 2 | 2 | 0.5 | 1 |
| **T033** | Pattern 3 | Gamma | 1 | 0 | 0 | 1 | 0 | 0 | 0 | Amygdala | 0 | 0 | 1 | 0 | 0 | 0 | 1 | 6 | 2 | 2.0 | 1 |
| **T051** | Pattern 3 | Gamma | 1 | NaN | 0 | 1 | 0 | 0 | 0 | Amygdala | 0 | 0 | 1 | 0 | 0 | 0 | 1 | 6 | 2 | IA | 0 |
| **T062** | Pattern 3 | Gamma | 1 | 0 | 1 | 2 | 1 | 1 | 0 | Amygdala | 0 | 0 | 1 | 0 | 0 | 0 | 1 | 4 | 1 | 2.8 | 0 |
| **T088** | Pattern 3 | Gamma | 1 | 0 | 0 | 3 | 0 | 0 | 0 | Amygdala | 0 | 0 | 1 | 0 | 0 | 0 | 1 | 3 | 2 | IA | 0 |
| **T118** | Pattern 3 | Gamma | 1 | 0 | 0 | 0 | 2 | 0 | 0 | Amygdala | 0 | 0 | 1 | 0 | 0 | 0 | 1 | 2 | 2 | IA | 0 |
| **T128** | Pattern 3 | Gamma | 1 | 0 | 0 | 0 | 2 | 0 | 0 | Amygdala | 0 | 0 | 1 | 0 | 0 | 0 | 1 | 3 | 2 | IA | 0 |
| **T133** | Pattern 3 | Gamma | 2 | NaN | 5 | 11 | 10 | 0 | 0 | Amygdala | 0 | 0 | 1 | 0 | 0 | 1 | 1 | 6 | 2 | IA | 0 |
| **T138** | Pattern 3 | Gamma | 2 | NaN | 4 | 5 | 3 | 1 | 0 | Amygdala | 0 | 0 | 1 | 0 | 0 | 1 | 0 | 3 | 2 | IA | 0 |
| **T003** | NaN | NaN | 0 | 0 | 0 | 0 | 0 | 0 | 0 | - | 0 | 0 | 0 | 0 | 0 | 0 | 0 | 6 | 2 | 0.9 | 0 |
| **T004** | NaN | NaN | 0 | 0 | 0 | 0 | 0 | 0 | 0 | - | 0 | 0 | 0 | 0 | 0 | 0 | 0 | 5 | 1 | 1.1 | 0 |
| **T006** | NaN | NaN | 0 | 0 | 0 | 0 | 0 | 0 | 0 | - | 0 | 0 | 0 | 0 | 0 | 0 | 0 | 6 | 2 | 0.5 | 0 |
| **T007** | NaN | NaN | 0 | 0 | 0 | 0 | 0 | 0 | 0 | - | 0 | 0 | 0 | 0 | 0 | 0 | 0 | 5 | 2 | 0.4 | 0 |
| **T008** | NaN | NaN | 0 | 0 | 0 | 0 | 0 | 0 | 0 | - | 0 | 0 | 0 | 0 | 0 | 0 | 0 | 2 | 0 | 1.1 | 0 |
| **T010** | NaN | NaN | 0 | 0 | 0 | 0 | 0 | 0 | 0 | - | 0 | 0 | 0 | 0 | 0 | 0 | 0 | 5 | 2 | 0.6 | 0 |
| **T013** | NaN | NaN | 0 | 0 | 0 | 0 | 0 | 0 | 0 | - | 0 | 0 | 0 | 0 | 0 | 0 | 0 | 2 | 0 | 0.6 | 0 |
| **T014** | NaN | NaN | 0 | 0 | 0 | 0 | 0 | 0 | 0 | - | 0 | 0 | 0 | 0 | 0 | 0 | 0 | 1 | 0 | 2.3 | 0 |
| **T019** | NaN | NaN | 0 | 0 | 0 | 0 | 0 | 0 | 0 | - | 0 | 0 | 0 | 0 | 0 | 0 | 0 | 5 | 1 | 0.3 | 0 |
| **T023** | NaN | NaN | 0 | 0 | 0 | 0 | 0 | 0 | 0 | - | 0 | 0 | 0 | 0 | 0 | 0 | 0 | 6 | 2 | 0.3 | 0 |
| **T024** | NaN | NaN | 0 | 0 | 0 | 0 | 0 | 0 | 0 | - | 0 | 0 | 0 | 0 | 0 | 0 | 0 | 5 | 2 | 0.7 | 0 |
| **T035** | NaN | NaN | 0 | 0 | 0 | 0 | 0 | 0 | 0 | - | 0 | 0 | 0 | 0 | 0 | 0 | 0 | 6 | 1 | 0.3 | 0 |
| **T037** | NaN | NaN | 0 | 0 | 0 | 0 | 0 | 0 | 0 | - | 0 | 0 | 0 | 0 | 0 | 0 | 0 | 3 | 2 | 0.8 | 0 |
| **T039** | NaN | NaN | 0 | 0 | 0 | 0 | 0 | 0 | 0 | - | 0 | 0 | 0 | 0 | 0 | 0 | 0 | 6 | 2 | 0.7 | 1 |
| **T043** | NaN | NaN | 0 | 0 | 0 | 0 | 0 | 0 | 0 | - | 0 | 0 | 0 | 0 | 0 | 0 | 0 | 6 | 2 | 0.5 | 0 |
| **T044** | NaN | NaN | 0 | 0 | 0 | 0 | 0 | 0 | 0 | - | 0 | 0 | 0 | 0 | 0 | 0 | 0 | 2 | 0 | 0.4 | 0 |
| **T045** | NaN | NaN | 0 | 0 | 0 | 0 | 0 | 0 | 0 | - | 0 | 0 | 0 | 0 | 0 | 0 | 0 | 3 | 1 | 2.5 | 0 |
| **T050** | NaN | NaN | 0 | 0 | 0 | 0 | 0 | 0 | 0 | - | 0 | 0 | 0 | 0 | 0 | 0 | 0 | 6 | 2 | 1.2 | 0 |
| **T052** | NaN | NaN | 0 | 0 | 0 | 0 | 0 | 0 | 0 | - | 0 | 0 | 0 | 0 | 0 | 0 | 0 | 1 | 1 | 4.2 | 0 |
| **T053** | NaN | NaN | 0 | 0 | 0 | 0 | 0 | 0 | 0 | - | 0 | 0 | 0 | 0 | 0 | 0 | 0 | 5 | 2 | 0.0 | 0 |
| **T054** | NaN | NaN | 0 | 0 | 0 | 0 | 0 | 0 | 0 | - | 0 | 0 | 0 | 0 | 0 | 0 | 0 | 6 | 2 | 0.3 | 0 |
| **T055** | NaN | NaN | 0 | 0 | 0 | 0 | 0 | 0 | 0 | - | 0 | 0 | 0 | 0 | 0 | 0 | 0 | 6 | 2 | 0.6 | 0 |
| **T065** | NaN | NaN | 0 | 0 | 0 | 0 | 0 | 0 | 0 | - | 0 | 0 | 0 | 0 | 0 | 0 | 0 | 2 | 0 | 0.6 | 0 |
| **T067** | NaN | NaN | 0 | 0 | 0 | 0 | 0 | 0 | 0 | - | 0 | 0 | 0 | 0 | 0 | 0 | 0 | 1 | 0 | 3.0 | 0 |
| **T069** | NaN | NaN | 0 | 0 | 0 | 0 | 0 | 0 | 0 | - | 0 | 0 | 0 | 0 | 0 | 0 | 0 | 1 | 0 | 1.2 | 0 |
| **T070** | NaN | NaN | 0 | 0 | 0 | 0 | 0 | 0 | 0 | - | 0 | 0 | 0 | 0 | 0 | 0 | 0 | 6 | 2 | 1.1 | 0 |
| **T072** | NaN | NaN | 0 | 0 | 0 | 0 | 0 | 0 | 0 | - | 0 | 0 | 0 | 0 | 0 | 0 | 0 | 2 | 0 | 0.5 | 0 |
| **T074** | NaN | NaN | 0 | 0 | 0 | 0 | 0 | 0 | 0 | - | 0 | 0 | 0 | 0 | 0 | 0 | 0 | 2 | 2 | 3.7 | 0 |
| **T075** | NaN | NaN | 0 | 0 | 0 | 0 | 0 | 0 | 0 | - | 0 | 0 | 0 | 0 | 0 | 0 | 0 | 1 | 0 | 2.1 | 0 |
| **T076** | NaN | NaN | 0 | 0 | 0 | 0 | 0 | 0 | 0 | - | 0 | 0 | 0 | 0 | 0 | 0 | 0 | 1 | 0 | 1.0 | 0 |
| **T077** | NaN | NaN | 0 | NaN | 0 | 0 | 0 | 0 | 0 | - | 0 | 0 | 0 | 0 | 0 | 0 | 0 | 4 | 2 | IA | 0 |
| **T079** | NaN | NaN | 0 | 0 | 0 | 0 | 0 | 0 | 0 | - | 0 | 0 | 0 | 0 | 0 | 0 | 0 | 1 | 0 | IA | 0 |
| **T080** | NaN | NaN | 0 | 0 | 0 | 0 | 0 | 0 | 0 | - | 0 | 0 | 0 | 0 | 0 | 0 | 0 | 3 | 0 | IA | 0 |
| **T081** | NaN | NaN | 0 | 0 | 0 | 0 | 0 | 0 | 0 | - | 0 | 0 | 0 | 0 | 0 | 0 | 0 | 4 | 2 | IA | 0 |
| **T084** | NaN | NaN | 0 | 0 | 0 | 0 | 0 | 0 | 0 | - | 0 | 0 | 0 | 0 | 0 | 0 | 0 | 3 | NaN | IA | 0 |
| **T085** | NaN | NaN | 0 | 0 | 0 | 0 | 0 | 0 | 0 | - | 0 | 0 | 0 | 0 | 0 | 0 | 0 | 1 | 0 | IA | 0 |
| **T086** | NaN | NaN | 0 | 0 | 0 | 0 | 0 | 0 | 0 | - | 0 | 0 | 0 | 0 | 0 | 0 | 0 | 2 | 0 | IA | 0 |
| **T089** | NaN | NaN | 0 | 0 | 0 | 0 | 0 | 0 | 0 | - | 0 | 0 | 0 | 0 | 0 | 0 | 0 | 4 | 2 | IA | 0 |
| **T094** | NaN | NaN | 0 | 0 | 0 | 0 | 0 | 0 | 0 | - | 0 | 0 | 0 | 0 | 0 | 0 | 0 | 3 | 0 | IA | 0 |
| **T095** | NaN | NaN | 0 | 0 | 0 | 0 | 0 | 0 | 0 | - | 0 | 0 | 0 | 0 | 0 | 0 | 0 | 1 | 0 | IA | 0 |
| **T097** | NaN | NaN | 0 | 0 | 0 | 0 | 0 | 0 | 0 | - | 0 | 0 | 0 | 0 | 0 | 0 | 0 | 4 | NaN | IA | 0 |
| **T098** | NaN | NaN | 0 | 0 | 0 | 0 | 0 | 0 | 0 | - | 0 | 0 | 0 | 0 | 0 | 0 | 0 | 1 | 0 | IA | 0 |
| **T099** | NaN | NaN | 0 | 0 | 0 | 0 | 0 | 0 | 0 | - | 0 | 0 | 0 | 0 | 0 | 0 | 0 | 3 | 0 | IA | 0 |
| **T100** | NaN | NaN | 0 | 0 | 0 | 0 | 0 | 0 | 0 | - | 0 | 0 | 0 | 0 | 0 | 0 | 0 | 2 | 0 | IA | 0 |
| **T101** | NaN | NaN | 0 | 0 | 0 | 0 | 0 | 0 | 0 | - | 0 | 0 | 0 | 0 | 0 | 0 | 0 | 4 | 2 | IA | 0 |
| **T102** | NaN | NaN | 0 | NaN | 0 | 0 | 0 | 0 | 0 | - | 0 | 0 | 0 | 0 | 0 | 0 | 0 | 1 | 0 | IA | 0 |
| **T103** | NaN | NaN | 0 | 0 | 0 | 0 | 0 | 0 | 0 | - | 0 | 0 | 0 | 0 | 0 | 0 | 0 | 2 | 0 | IA | 0 |
| **T104** | NaN | NaN | 0 | 0 | 0 | 0 | 0 | 0 | 0 | - | 0 | 0 | 0 | 0 | 0 | 0 | 0 | 1 | 0 | IA | 0 |
| **T105** | NaN | NaN | 0 | 0 | 0 | 0 | 0 | 0 | 0 | - | 0 | 0 | 0 | 0 | 0 | 0 | 0 | 1 | 1 | IA | 0 |
| **T106** | NaN | NaN | 0 | NaN | 0 | 0 | 0 | 0 | 0 | - | 0 | 0 | 0 | 0 | 0 | 0 | 0 | 4 | 0 | IA | 0 |
| **T108** | NaN | NaN | 0 | 0 | 0 | 0 | 0 | 0 | 0 | - | 0 | 0 | 0 | 0 | 0 | 0 | 0 | 3 | 2 | IA | 0 |
| **T111** | NaN | NaN | 0 | 0 | 0 | 0 | 0 | 0 | 0 | - | 0 | 0 | 0 | 0 | 0 | 0 | 0 | 1 | 0 | IA | 0 |
| **T114** | NaN | NaN | 0 | 0 | 0 | 0 | 0 | 0 | 0 | - | 0 | 0 | 0 | 0 | 0 | 0 | 0 | 3 | 2 | IA | 0 |
| **T116** | NaN | NaN | 0 | NaN | 0 | 0 | 0 | 0 | 0 | - | 0 | 0 | 0 | 0 | 0 | 0 | 0 | 3 | 2 | IA | 0 |
| **T117** | NaN | NaN | 0 | 0 | 0 | 0 | 0 | 0 | 0 | - | 0 | 0 | 0 | 0 | 0 | 0 | 0 | 5 | 2 | IA | 0 |
| **T119** | NaN | NaN | 0 | 0 | 0 | 0 | 0 | 0 | 0 | - | 0 | 0 | 0 | 0 | 0 | 0 | 0 | 1 | 0 | IA | 0 |
| **T120** | NaN | NaN | 0 | 0 | 0 | 0 | 0 | 0 | 0 | - | 0 | 0 | 0 | 0 | 0 | 0 | 0 | 3 | 0 | IA | 0 |
| **T121** | NaN | NaN | 0 | 0 | 0 | 0 | 0 | 0 | 0 | - | 0 | 0 | 0 | 0 | 0 | 0 | 0 | 2 | 2 | IA | 0 |
| **T122** | NaN | NaN | 0 | 0 | 0 | 0 | 0 | 0 | 0 | - | 0 | 0 | 0 | 0 | 0 | 0 | 0 | 2 | 0 | IA | 0 |
| **T123** | NaN | NaN | 0 | 0 | 0 | 0 | 0 | 0 | 0 | - | 0 | 0 | 0 | 0 | 0 | 0 | 0 | 3 | 0 | IA | 0 |
| **T124** | NaN | NaN | 0 | 0 | 0 | 0 | 0 | 0 | 0 | - | 0 | 0 | 0 | 0 | 0 | 0 | 0 | 2 | 0 | IA | 0 |
| **T125** | NaN | NaN | 0 | NaN | NaN | 0 | 0 | 0 | 0 | - | 0 | 0 | 0 | 0 | 0 | 0 | 0 | 5 | 2 | IA | 0 |
| **T126** | NaN | NaN | 0 | 0 | 0 | 0 | 0 | 0 | 0 | - | 0 | 0 | 0 | 0 | 0 | 0 | 0 | 6 | 2 | IA | 0 |
| **T127** | NaN | NaN | 0 | 0 | 0 | 0 | 0 | 0 | 0 | - | 0 | 0 | 0 | 0 | 0 | 0 | 0 | 1 | 0 | IA | 0 |
| **T131** | NaN | NaN | 0 | 0 | 0 | 0 | 0 | 0 | 0 | - | 0 | 0 | 0 | 0 | 0 | 0 | 0 | 6 | 2 | IA | 0 |
| **T132** | NaN | NaN | 0 | NaN | 0 | 0 | 0 | 0 | 0 | - | 0 | 0 | 0 | 0 | 0 | 0 | 0 | 1 | 0 | IA | 0 |
| **T134** | NaN | NaN | 0 | 0 | 0 | 0 | 0 | 0 | 0 | - | 0 | 0 | 0 | 0 | 0 | 0 | 0 | 6 | 2 | IA | 0 |
| **T135** | NaN | NaN | 0 | 0 | 0 | 0 | 0 | 0 | 0 | - | 0 | 0 | 0 | 0 | 0 | 0 | 0 | 3 | NaN | IA | 0 |
| **T136** | NaN | NaN | 0 | NaN | 0 | 0 | 0 | 0 | 0 | - | 0 | 0 | 0 | 0 | 0 | 0 | 0 | 5 | 2 | IA | 1 |
| **T137** | NaN | NaN | 0 | 0 | 0 | 0 | 0 | 0 | 0 | - | 0 | 0 | 0 | 0 | 0 | 0 | 0 | 5 | 2 | IA | 0 |
| **T139** | NaN | NaN | 0 | 0 | 0 | 0 | 0 | 0 | 0 | - | 0 | 0 | 0 | 0 | 0 | 0 | 0 | 1 | 0 | IA | 0 |
| **T161** | NaN | NaN | 0 | 0 | 0 | 0 | 0 | 0 | 0 | - | 0 | 0 | 0 | 0 | 0 | 0 | 0 | 3 | 0 | 0.7 | 0 |
| **T172** | NaN | NaN | 0 | 0 | 0 | 0 | 0 | 0 | 0 | - | 0 | 0 | 0 | 0 | 0 | 0 | 0 | 1 | 2 | 1.9 | 0 |
| **T173** | NaN | NaN | 0 | 0 | 0 | 0 | 0 | 0 | 0 | - | 0 | 0 | 0 | 0 | 0 | 0 | 0 | 6 | 2 | 0.3 | 0 |
| **T174** | NaN | NaN | 0 | 0 | 0 | 0 | 0 | 0 | 0 | - | 0 | 0 | 0 | 0 | 0 | 0 | 0 | 6 | 2 | 1.0 | 0 |
| **T179** | NaN | NaN | 0 | 0 | 0 | 0 | 0 | 0 | 0 | - | 0 | 0 | 0 | 0 | 0 | 0 | 0 | 5 | 2 | 3.3 | 1 |
| **T180** | NaN | NaN | 0 | 0 | 0 | 0 | 0 | 0 | 0 | - | 0 | 0 | 0 | 0 | 0 | 0 | 0 | 5 | 1 | 0.6 | 1 |
| **Notes and Abbreviations**: NaN-not relevant or not available; IA-information available at autopsy; pTDP-pTDP IHC severity (0-4); Cog (0, normal; 1, MCI; 2, dementia); Interval-time between last assessment and autopsy (years); HS-hippocampal sclerosis (H&E). | | | | | | | | | | | | | | | | | | | | | |
