# Supplementary figures and images for: Patterns of amygdala region pathology in LATE-NC: subtypes that differ with regard to TDP-43 histopathology, genetic risk factors, and comorbid pathologies
Source: Acta Neuropathol. 2022 Apr 2;143(5):531–45. doi: 10.1007/s00401-022-02416-5 (PMC9038848; doi:10.1007/s00401-022-02416-5)

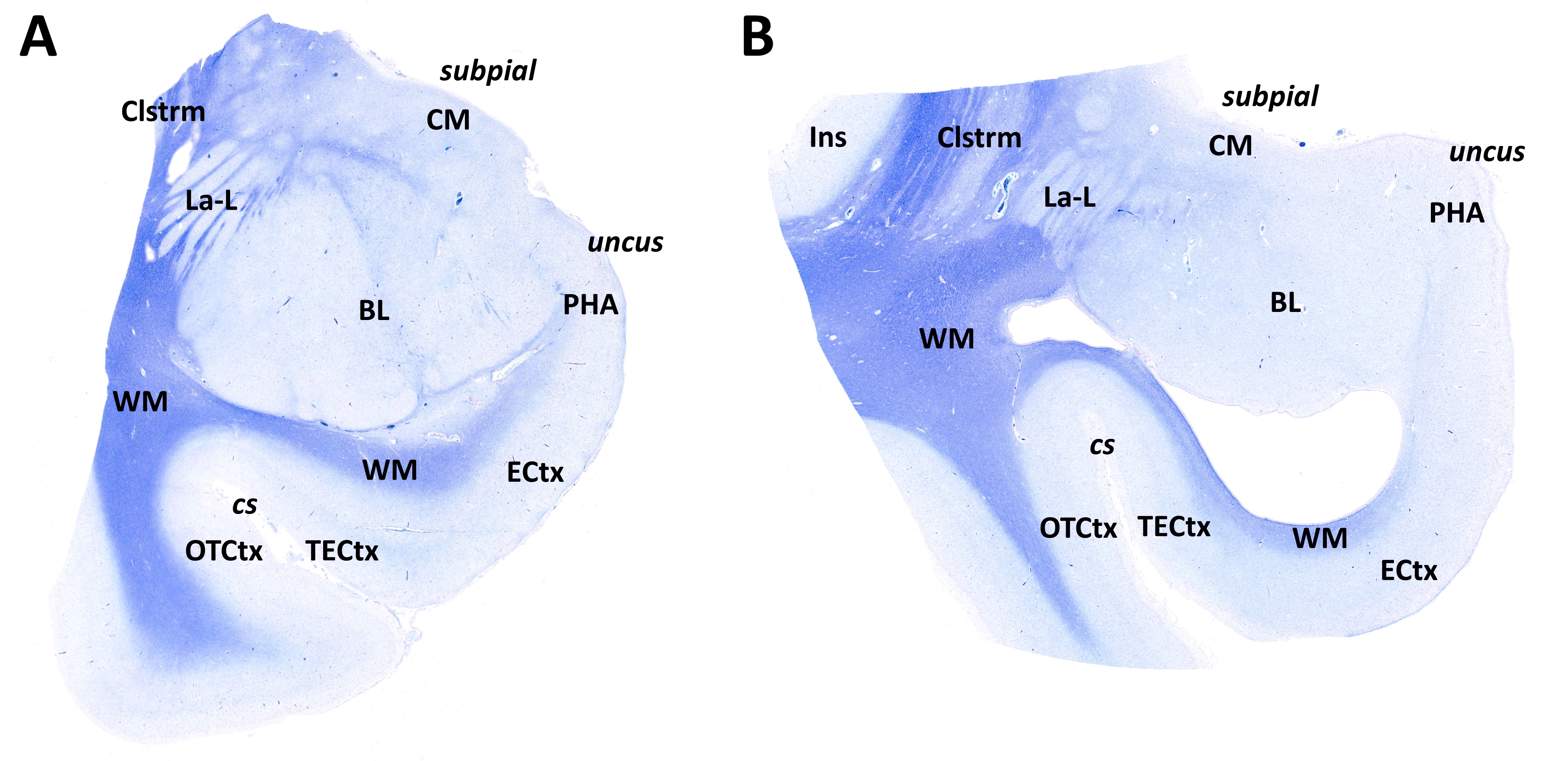

Supplement: Supplementary file 3 — Supplementary file3 Supplemental Figure 1. Scanned LFB/Nissl sections of two study samples, without (A) and with (B) atrophy in the amygdala region. The major study ROIs are shown, as also depicted in the schematic of Figure 1. (TIF 8410 KB) [file 401_2022_2416_MOESM3_ESM.tif]

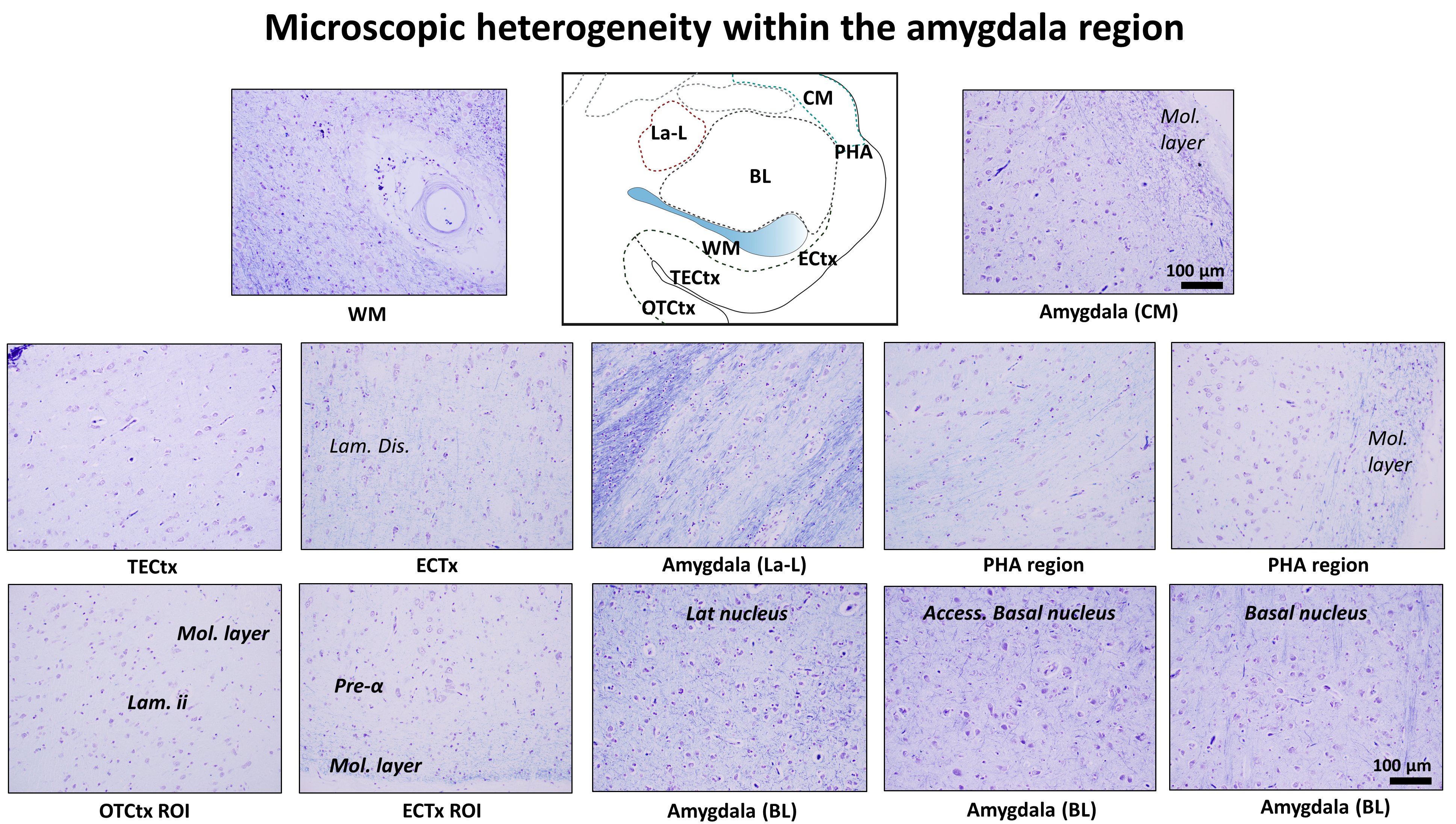

Supplement: Supplementary file 4 — Supplementary file4 Supplemental Figure 2. Microscopic heterogeneity within the amygdala region. LFB/Nissl images of representative fields as shown in the schematic at top center (all photomicrographs shown are at 200x magnification and the scale bar in the top right and lower right panels applies to all panels). Note the heterogeneity of cell density, cell orientation and cell size, even with components of “amygdala”, including ventral cortical nucleus (labeled “CM”), basal (BL) and accessory basal (AB) nuclei, lateral nucleus (Lat) and its lateral division (La-L). Because of the variability across cases, BL, AB, and Lat were grouped into a single ROI (“BL ROI”), and VCo and the parahippocampal-amygdaloid transition area (PHA) was grouped into a single ROI (“CM”). The central and medial nuclei were not incorporated into the CM nucleus because they were not consistent present across all samples; when present, these were never a significant site of early pTDP-43 pathology. (TIF 11119 KB) [file 401_2022_2416_MOESM4_ESM.tif]

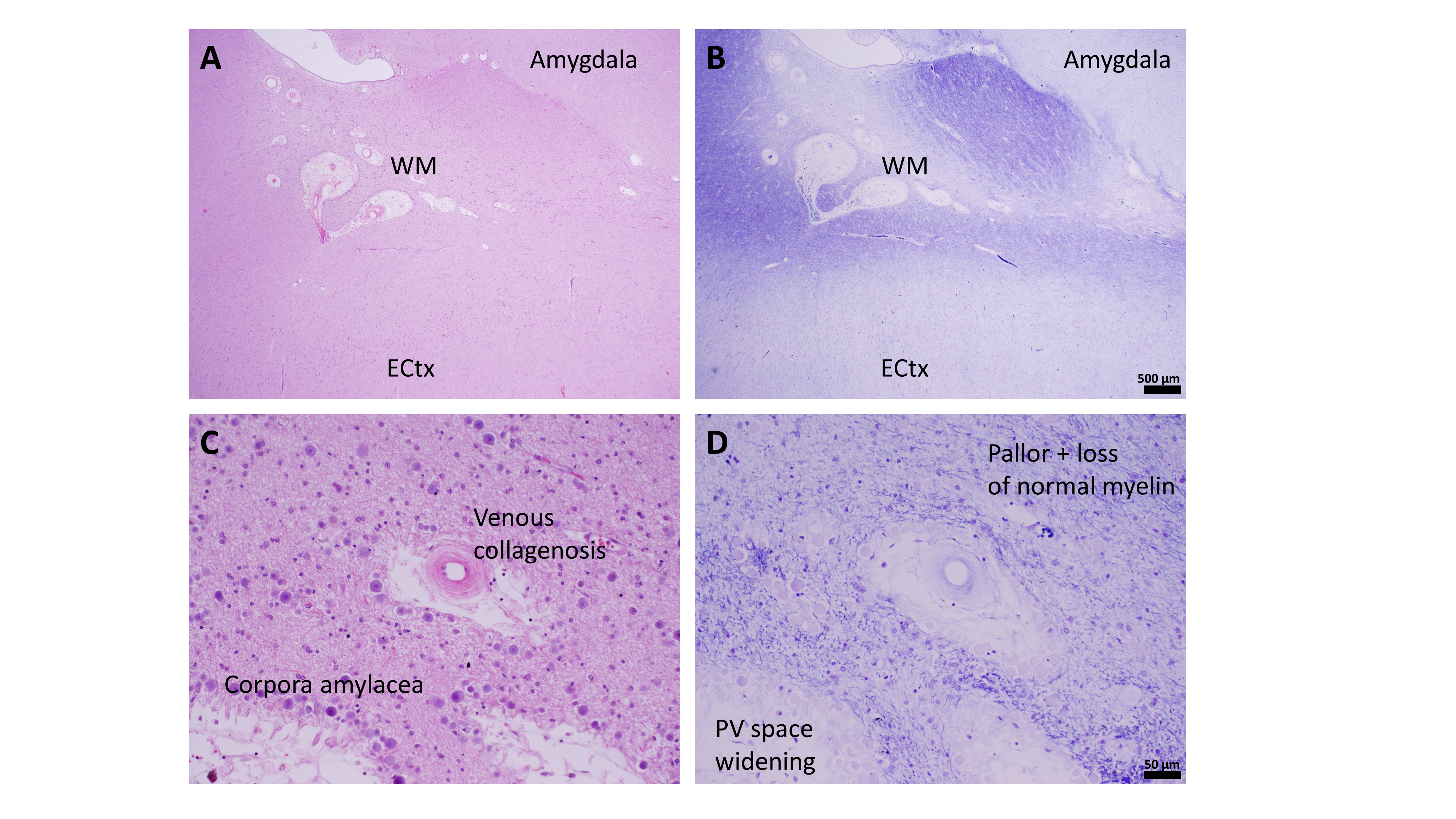

Supplement: Supplementary file 5 — Supplementary file5 Supplemental Figure 3. Subamygdaloid white matter and surrounding structures. H&E (panels A, C) and LFB/Nissl (panels B, D) stains show subamygdaloid white matter (“WM”), which is positioned between the basolateral division of amygdala (“Amygdala”) and entorhinal cortex (“ECtx”). This is a common site for conspicuous vascular pathologies, including venous collagenosis (panel C) and perivascular space widening (panels C, D). Corpora amylacea may also be abundant in this region (panel C). One of the morphologic measures in the amygdala region of this study was the combination of rarefied white matter (H&E) with loss of myelin (LFB), venous collagenosis, perivascular space widening and abundant corpora amylacea (scored as present or absent). The sample shown here demonstrates an example of the pattern being “present”. (TIF 11156 KB) [file 401_2022_2416_MOESM5_ESM.tif]

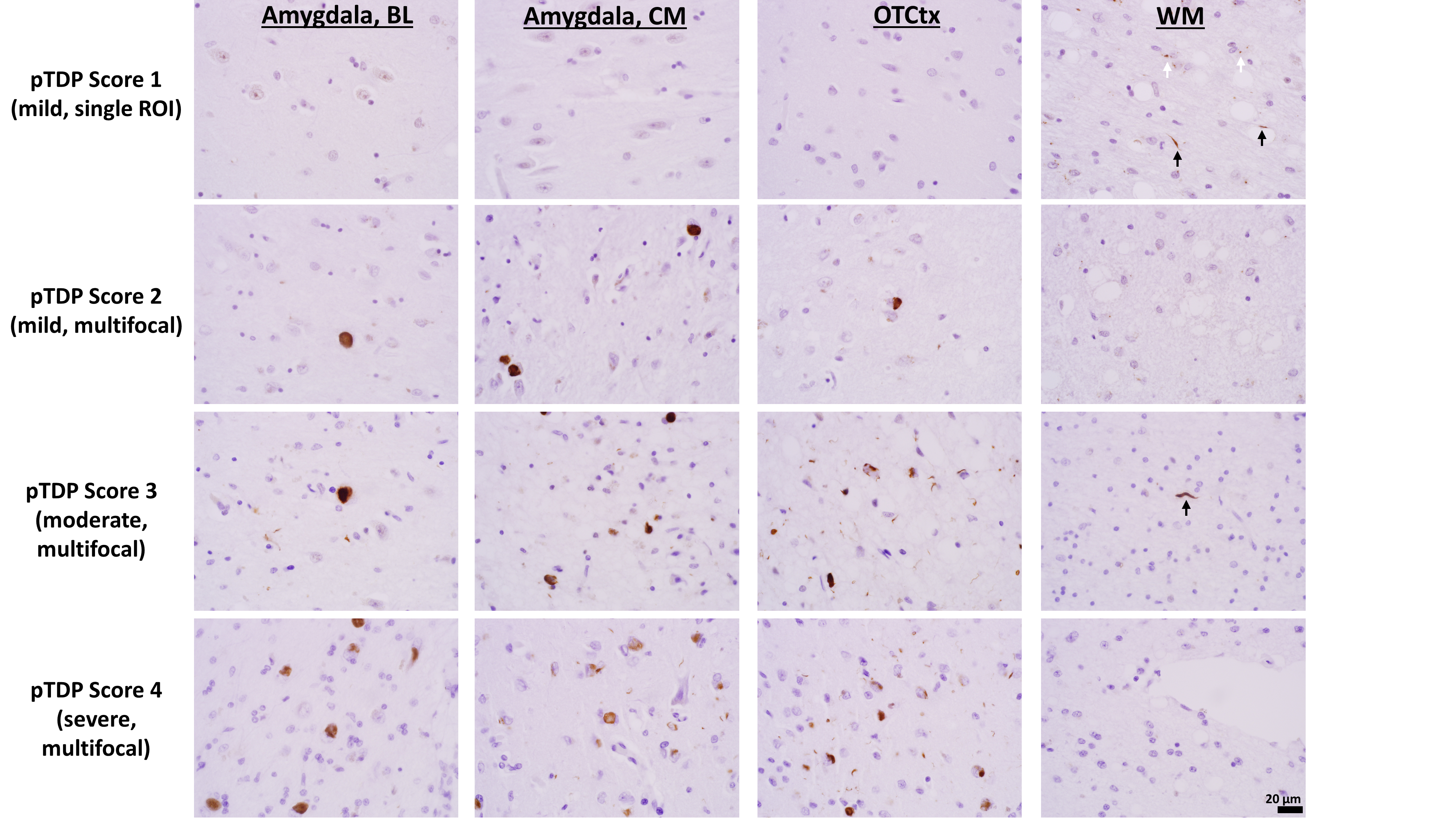

Supplement: Supplementary file 6 — Supplementary file6 Supplemental Figure 4. Scoring of pTDP-43 inclusion density in amygdala region. pTDP-43 staining in four representative ROIs are shown in four different amygdalae with LATE-NC (one subject is shown per row). ROIs shown include basolateral nuclear group of amygdala (“BL”), the parahippocampal–amygdaloid transition zone of the CM ROI, layer 2 of the occipitotemporal cortex (“OTCtx”), and the subamygdaloid white matter (“WM”) that is positioned between amygdala and entorhinal cortex. The definitions of scores 0–4 are further defined in the Methods and considered all study ROIs, not just those depicted here. (TIF 8320 KB) [file 401_2022_2416_MOESM6_ESM.tif]

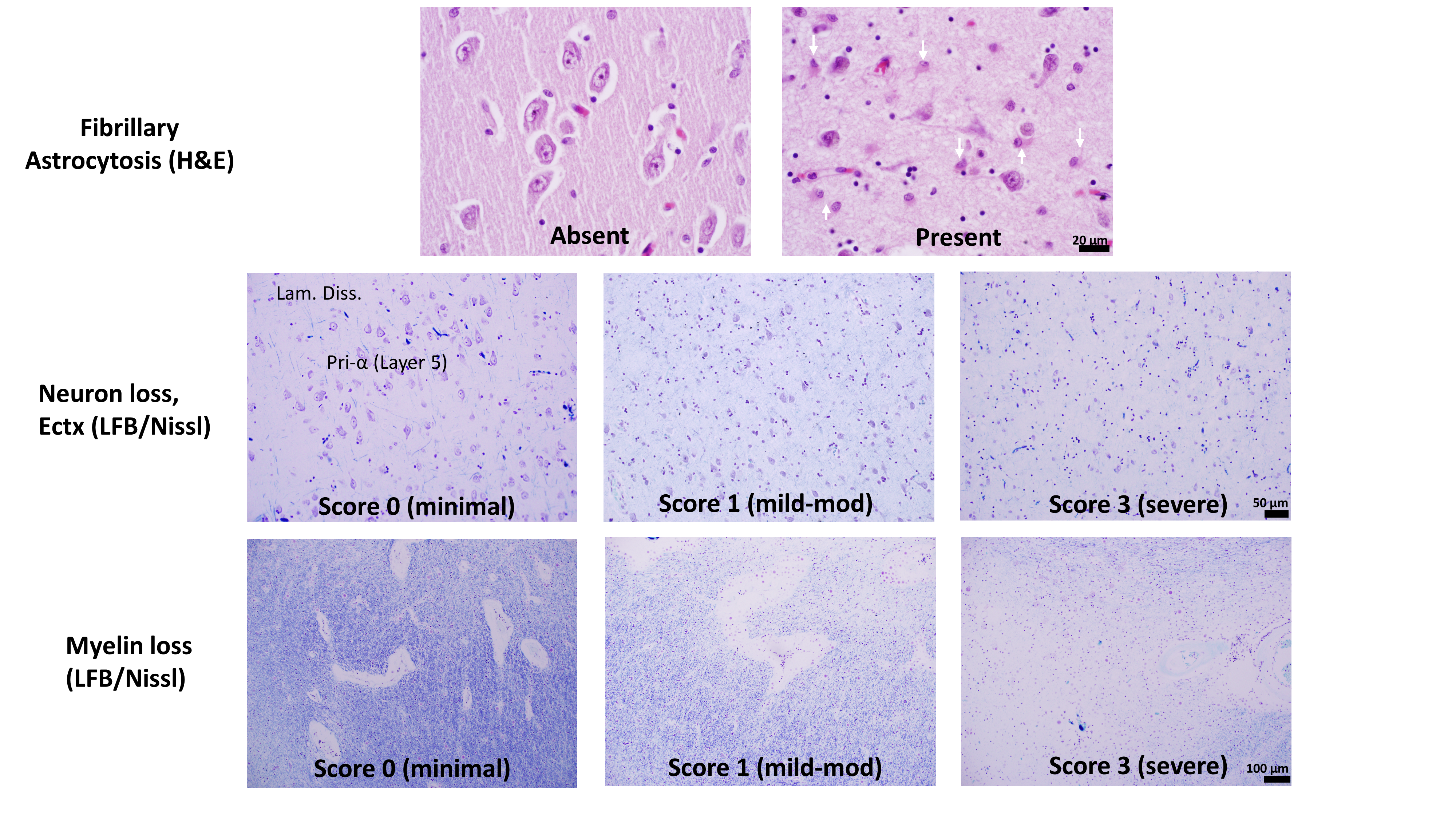

Supplement: Supplementary file 7 — Supplementary file7 Supplemental Figure 5. Additional pathologic measures in the amygdala region. Pathologies assessed in the amygdala region, blinded to pTDP-43 status, included fibrillary astrocytosis (H&E) (top row), assessed as present/absent, as well as three-tiered scales to assess neuronal loss in entorhinal cortex (Nissl/LFB) (middle row) and myelin loss (bottom row), particularly in white matter between amygdala and entorhinal cortex. Top row images are from the deep laminae of entorhinal cortex and conspicuous reactive astrocytes are indicated by white arrows. Middle row images are centered on the layer V (pri-α) of entorhinal cortex, and this layer as well as the overlying lamina dissecans (“Lam. Diss”) are labeled. Scale bars for right-most images apply to all images on that row. (TIF 10386 KB) [file 401_2022_2416_MOESM7_ESM.tif]

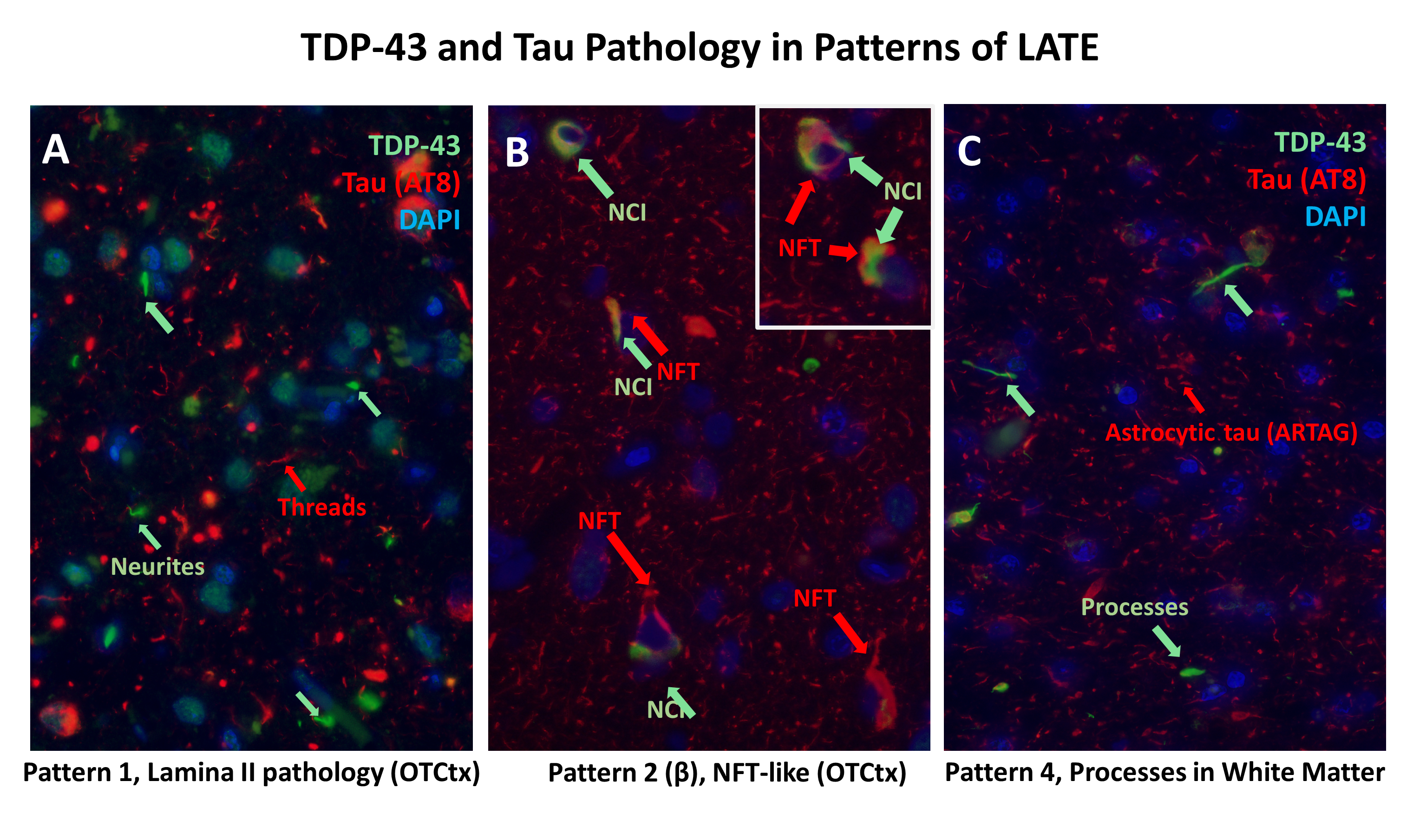

Supplement: Supplementary file 8 — Supplementary file8 Supplemental Figure 6. Overlapping and non-overlapping TDP-43 and Tau pathologies in LATE-NC. (A) The short non-tapering neurites in lamina II that characterized Pattern 1 (type-α) did not co-localize with tau-positive pathology. (B) In contrast, TDP-43 and Tau co-localized in the same neurons and even cytoplasmic foci in this example of a Pattern 2 (type-β). (C) LATE-NC was significantly associated with amygdala region ARTAG, although the TDP-43 processes of Pattern 4 and tau-positive ARTAG did not co-localize. (TIF 6085 KB) [file 401_2022_2416_MOESM8_ESM.tif]

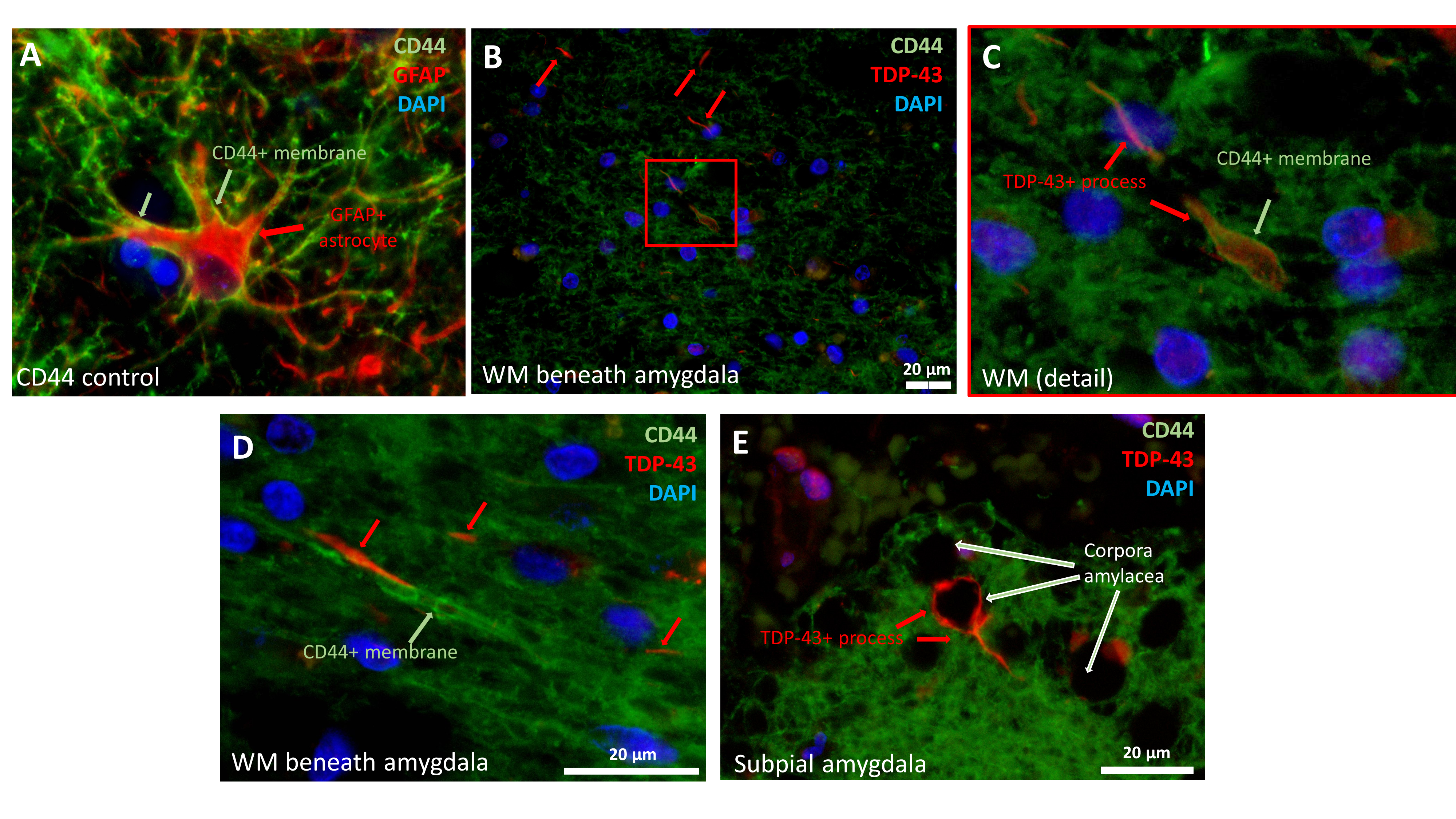

Supplement: Supplementary file 9 — Supplementary file9 Supplemental Figure 7. CD44 and TDP-43 labeling in WM and subpial amygdala. CD44 is a non-specific marker, that can be used to highlight astrocytic membranes, as shown in the control CD44-GFAP image in panel A. In WM (B, C, and D), CD44 staining and TDP-43 stains show the proximity of TDP-43+ processes and CD44+ membranes, suggesting some of this TDP-43 pathology may occur within astrocytes. Note that in panel D (subpial region of amygdala), TDP-43+ process wrap around corpora amylacea, appearing as a negative space in the image surrounded by CD44+ processes. Panel C is enlarged from the region-of-interest in panel B. Panels D and E are enlarged, and a scale bar is provided for both. (TIF 10262 KB) [file 401_2022_2416_MOESM9_ESM.tif]

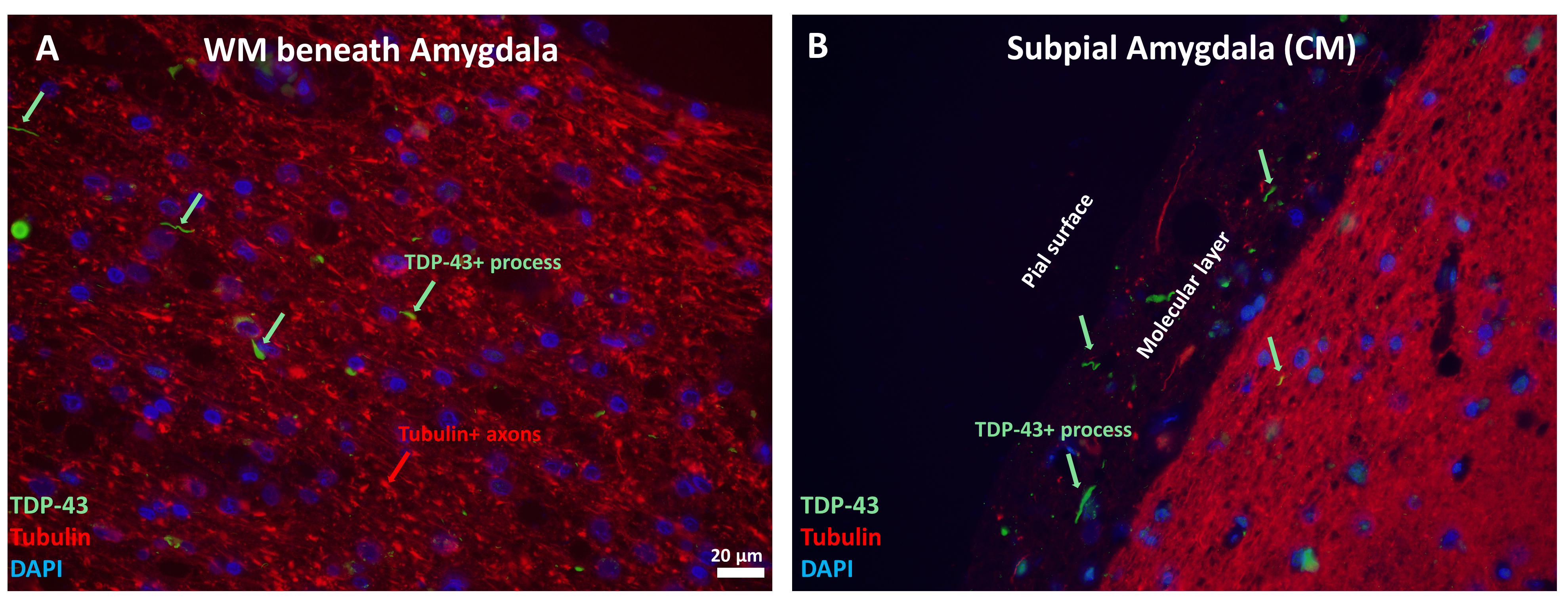

Supplement: Supplementary file 10 — Supplementary file10 Supplemental Figure 8. TDP-43-positive processes and axonal markers. Select samples with Pattern 4 (δ), characterized by TDP-43+ processes, were also investigated using double labeling for TDP-43 and three neuronal and axonal markers. As shown in WM (A) and subpial amygdala (B), no overlap was seen. The possibility that some of this pathology is neuritic/axonal cannot entirely be excluded, but better co-localization was seen in foci of dense CD44 staining (Supplemental Fig. 7) and GFAP labeling (not shown). Scale bar applies to both images. (TIF 8378 KB) [file 401_2022_2416_MOESM10_ESM.tif]

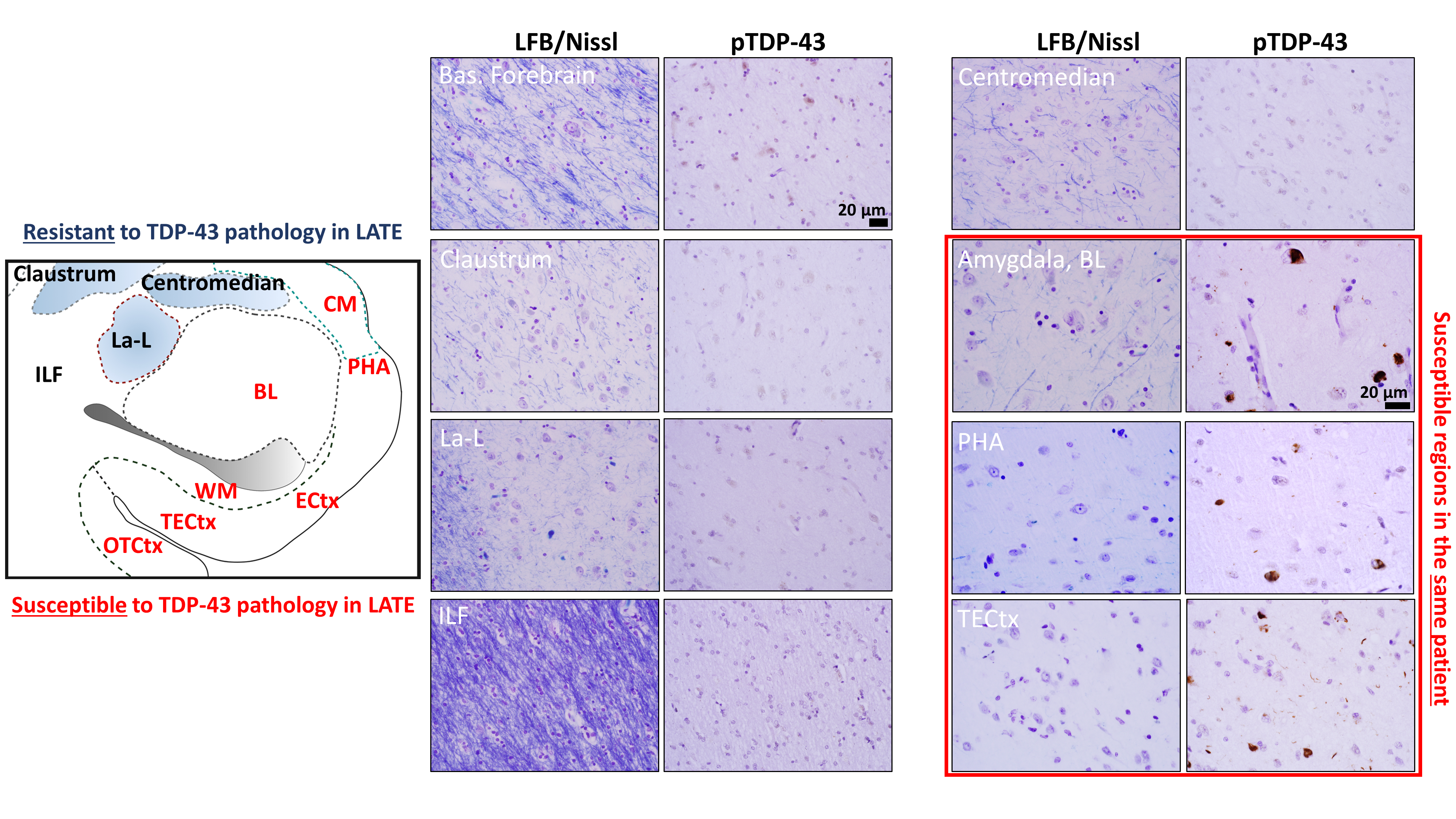

Supplement: Supplementary file 11 — Supplementary file11 Supplemental Figure 9. Regions resistant to pTDP-43 pathology in LATE-NC. Paired LFB/Nissl and pTDP-43 stains in ROIs in a single study subject with mild LATE-NC in the amygdala region. Three ROIs frequently positive in LATE-NC are highlighted with a red outline at the bottom right (images taken at 600x and the scale bar for “Amygdala, BL” applies to all three sets of ROIs). In contrast, negative ROIs dorsally and laterally are shown, including ventrolateral extent of basal forebrain, ventral extension of claustrum (near La-L), the lateral subdivision of the lateral nucleus of amygdala (La-L), the inferior longitudinal fasciculus (ILF) (in contrast, subamygdaloid WM was positive in this subject), and centromedian nuclear complex. This subject is representative of many cases with this medial to ventrolateral and lateral gradient of pTDP-43 pathology in LATE-NC. (TIF 8882 KB) [file 401_2022_2416_MOESM11_ESM.tif]
